# m^6^A RNA methylation modulates IFN-γ-stimulated intestinal epithelial cell-intrinsic antiparasitic defense

**DOI:** 10.1371/journal.ppat.1014442

**Published:** 2026-07-20

**Authors:** Chansorena Pok, Ai-Yu Gong, Marion L. Graham, Shuhong Wang, Silu Deng, Zinat Sharmin, Eugene Lu, Guoqing Lu, Chuan He, Xian-Ming Chen

**Affiliations:** 1 Department of Microbial Pathogens and Immunity, Rush University Medical Center, Chicago, Illinois, United States of America; 2 Department of Biology, School of Interdisciplinary Informatics, University of Nebraska at Omaha, Omaha, Nebraska, United States of America; 3 Department of Biochemistry, University of Chicago, Chicago, Illinois, United States of America; University of Utah, UNITED STATES OF AMERICA

## Abstract

*N*^6^-methyladenosine (m^6^A) RNA methylation is one of the most prevalent reversible post-transcriptional RNA modifications and has been recognized as a crucial regulator of host immune responses. Intestinal epithelial cells (IECs) constitute an important component of gastrointestinal mucosal immunity. Interferons (IFNs) play a central role in maintaining intestinal homeostasis, and m^6^A methylation status influences IFN-mediated cell-intrinsic defense. In this study, we investigated the potential role of m^6^A RNA modifications in IFN-γ-stimulated IEC-intrinsic defense. We observed significant alterations in the topology of the m^6^A mRNA methylome in murine IECs following IFN-γ stimulation. A subset of IFN-γ-stimulated immune gene transcripts exhibited increased m^6^A RNA methylation, including several members of the immunity-related GTPase family M (IRGM) genes. In addition, IFN-γ-responsive long non-coding RNAs may modulate the m^6^A methylation levels of multiple IFN-γ-stimulated immune transcripts. Enhanced m^6^A methylation of the *Irgm2/3* transcripts was associated with strengthened cell-intrinsic defense against infection by the protozoan parasite *Cryptosporidium*. Notably, *Cryptosporidium* infection altered the host m^6^A mRNA methylome in IECs, thereby counteracting the IFN-γ-mediated defense response. Although the RNA levels of *Irgm2/3* genes were upregulated, their m^6^A RNA methylation levels and protein expression were reduced in infected cells. This effect was associated with host delivery of dsRNAs derived from *Cryptosporidium parvum virus 1, a virus harbored in the parasite*. Collectively, our findings suggest that m^6^A methylation of RNA transcripts enhances IFN-γ-mediated IEC-intrinsic antiparasitic defense, while *Cryptosporidium* has evolved mechanisms to evade this response by suppressing m^6^A RNA methylation of IFN-γ-stimulated immune genes.

## Author summary

Cell-intrinsic defense refers to the ability of an individual host cell to autonomously eliminate invading microbes, a process that is especially important for controlling intracellular pathogens. The cytokine IFN-γ is a key regulator of epithelial cell–intrinsic defense. RNA modifications have emerged in recent years as a critical component of cell biology, including the regulation of host innate immune defense. The most common RNA modification in mRNA is N^6^ methyladenosine (m^6^A). The obligate intracellular parasite *Cryptosporidium* establishes an intracellular but extracytoplasmic infection by forming a membrane-bound vacuole on the apical surface of intestinal epithelial cells (IECs). Because infection is largely restricted to IECs, cell–intrinsic defense mechanisms—particularly those induced by IFN-γ—are essential for clearing the parasite. In this study, we investigated the role of m⁶A-mediated RNA methylation in regulating the IFN-γ–induced, cell-intrinsic anti-*Cryptosporidium* response in IECs. Our findings indicate that m⁶A-mediated post-transcriptional gene regulation is a key determinant of IFN-γ–induced epithelial antiparasitic defense. We further show that *C. parvum* has evolved mechanisms to evade host immunity by suppressing m⁶A methylation of host RNAs. These results provide new mechanistic insights into IEC-intrinsic immunity and may inform the development of novel therapeutic strategies.

## Introduction

Cell-intrinsic immunity (also called cell-autonomous immunity) is the ability of a host cell to eliminate an invasive/intracellular infectious agent at the cellular level. It serves as the first line of host defense against intracellular pathogens [1]. Intestinal epithelial cells (IECs) possess the necessary molecular machinery to mount a cell-intrinsic defense response, constituting an important component of gastrointestinal mucosal immunity. Ligation of their pattern recognition receptors leads to the upregulation of antimicrobial factors, secretion of cytokines and chemokines, and conditioning of immune cells for direct antimicrobial action or instruction of adaptive immune responses [2]. On the other hand, IECs serve as targets of mucosal immune mediators released from immune cells residing at the gastrointestinal mucosa [3]. One group of such cytokines is the family of interferons (IFNs). The IFN family can be classified into three main types: type I (e.g., IFN-α and IFN-β), type II (IFN-γ), and type III (IFN-λ family) [4]. Growing evidence supports the essential role of IFN-γ in antibacterial and antiparasitic immunity, whereas type I and type III IFNs are key to antiviral immunity [5, 6]. The canonical IFN-γ signaling utilizes the JAK/STAT signaling to activate STAT1, resulting in the formation and nuclear translocation of active STAT1 homodimers and transcription of IFN-stimulated genes (ISGs) [7, 8]. Many ISGs are primary effectors of the innate immune response, such as Mx1/MxA, the interferon-Induced proteins with tetratricopeptide repeats (IFIT) family, and the immunity-related GTPase family M (IRGM) genes [9–11]. The protein products of these genes can target different stages of a pathogen’s life cycle [9–11], regulate autophagy formation [12–14], and have been identified as cornerstones of IFN-γ-mediated responses to intracellular pathogens [15].

RNA modifications have gained traction in the past decade as a crucial element in eukaryotic biological processes. Among the most prevalent modifications occurring in approximately 25% of transcripts at the genome level in eukaryotes is m^6^A methylation [16]. *N*^6^-methyladenosine (m^6^A) is a post-transcriptional modification that can influence aspects of RNA metabolism, such as mRNA degradation, RNA splicing, mRNA stabilization, and translation efficiency [17]. m^6^A dynamics and functions are executed by three groups of proteins: methyltransferases, demethylases, and m^6^A-binding proteins [18]. m^6^A is installed on RNA molecules by the methyltransferase complex, consisting of methyltransferase-like 3 (METTL3) and METTL14 and their cofactor the WT1 Associated Protein [19]. Structural studies revealed that METTL3 primarily functions as the catalytic core, while METTL14 serves as an RNA-binding platform [20, 21]. m^6^A is mainly removed by the AlkB homolog H5 and fat mass and obesity-associated protein [22]. The m^6^A modification regulates RNA splicing, translocation, stability, and translation [23–25]. It has been well-recognized as a crucial regulator in T cell homeostasis, inflammation, and immune response [24, 25]. Selectively altering m^6^A levels, along with other immunotherapies, may be effective management strategies for a variety of immunological diseases.

*Cryptosporidium*, a coccidian parasite and an NIAID Category B priority pathogen, infects the gastrointestinal epithelium and is a leading cause of infectious diarrhea and diarrheal-related death in children worldwide [26]. The infection can also cause a life-threatening diarrheal disease in AIDS patients [27]. *Cryptosporidium* attaches to the apical membrane surface of epithelial cells and forms an intracellular but extracytoplasmic vacuole in which the organism remains [28]. Thus, cell-intrinsic defense is critical to the host’s defense against *Cryptosporidium* infection [29], providing an ideal model to explore intestinal epithelial cell-intrinsic immunity [30]. IFN-γ is key to epithelial cell-intrinsic anti-*Cryptosporidium* defense [5,6,31].

To counteract host defense, *Cryptosporidium* has evolved mechanisms to effectively dysregulate the IFN-γ signaling pathway [32]; thus, it can survive host immune attack during the early stages of infection [33, 34]. The *Cryptosporidium parvum virus 1* (CSpV1), a non-enveloped RNA virus of the *Partitiviridae* family, infects *C. parvum* and other *Cryptosporidium* spp. including *C. hominis* [35]. Its genome comprises two distinct double-stranded RNAs (dsRNAs), sized 1,836 bp (CSpV1-dsRdRp) and 1,510 bp (CSpV1-dsCA) [36]. The CSpV1-dsRNAs are not 5’-capped at either end and unlikely to be 3’-polyadenylylated [37]. CSpV1 is likely to be regularly transmitted only by intracellular routes, as it lacks the machinery for cell entry [38]. Importantly, the fecundity of *C. parvum* is correlated with its intracellular CSpV1 levels [39]. In our recent studies [40], we demonstrated that CSpV1-dsRNAs are present within infected host cells and are associated with attenuation of IFN-γ–mediated antiparasitic defense in IECs.

In this study, we demonstrated that IFN-γ stimulation causes significant alterations in the RNA m^6^A landscape of murine IECs, involving Mettl3/14 and long non-coding RNAs (lncRNAs). Increased m^6^A methylation of *Irgm2/3* RNAs following IFN-γ stimulation is associated with enhanced cell-intrinsic defense against *Cryptosporidium* infection. In contrast, *Cryptosporidium* infection is associated with reduced m^6^A methylation of *Irgm2/3* RNAs in infected IECs, potentially mediated by parasite-derived CSpV1-dsRNAs, coinciding with attenuation of IFN-γ-stimulated epithelial cell-intrinsic defense. Overall, our findings suggest that m^6^A-mediated post-transcriptional gene regulation may be a key determinant of IFN-γ-mediated epithelial antiparasitic defense, and that *C. parvum* may, at least in part, evade host immunity by suppressing m^6^A methylation of host RNAs.

## Results

### RNA-Seq transcriptomics and m^6^A mRNA topology of IECs following IFN-γ stimulation

**Fig 1:**
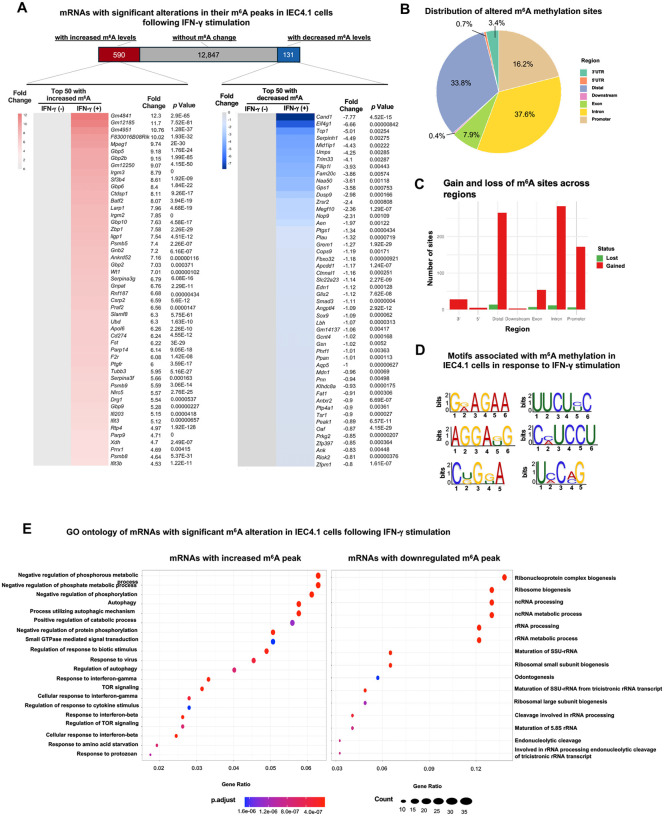
m^6^A topology of intestinal epithelial cells following IFN-γ stimulation. IEC 4.1 cells were treated with IFN-γ (10 ng/mL, 6 h). Total RNAs were collected and processed for m^6^A methylated RNA immunoprecipitation sequence (MeRIP-seq). **(A)** IFN-γ stimulation resulted in significant alterations in m^6^A peaks of mRNAs in the transcriptome. Bar is a schematic representation of the number of mRNAs with either unchanged or altered m^6^A levels after IFN-γ treatment. Heat map depicts top 50 mRNAs with increased or decreased m^6^A peaks. **(B)** Pie chart depicting overall distribution of m^6^A methylation sites across genomic transcript regions. RefSeq-based coordinates from MeRIP-Seq were converted to genomic coordinates using biomaRt. ChIPseeker was used to assign m^6^A peaks to genomic regions. **(C)** Bar graph depicting gain and loss of m^6^A methylation sites within each transcript region, determined by fold change status. Genomic annotations were derived from ChIPseeker. **(D)** Motif enrichment analysis showing the top three sequence motifs associated with newly emerged and lost m^6^A peaks in IEC4.1 cells following IFN-γ stimulation. **(E)** Gene ontology (GO) analysis depicting biological processes of mRNAs with increased or decreased m^6^A peaks in IFN-γ-treated-IEC 4.1 cells. *p* values were calculated based on Kolmogorov-Smirnov test and adjusted by Benjamini-Hochberg method. Data from **A**-**E** were derived from three biological replicates for each group (untreated and IFN-γ treated).

We first examined the landscape of the m^6^A mRNA methylome in IECs upon IFN-γ stimulation by performing m^6^A methylated RNA immunoprecipitation sequence (MeRIP-seq). IEC4.1 cells, a transformed but non-tumorigenic intestinal epithelial cell line derived from neonatal mice (5–7 days old) [41], were treated with IFN-γ (10 ng/ml) for 6 h. Total mRNA was then collected and processed for MeRIP-seq as previously reported [42]. We compared the abundance and distribution of m^6^A peaks on mRNAs between untreated control and IFN-γ-treated cells. IFN-γ stimulation resulted in significant alterations in m^6^A peaks across 721 mRNAs in the transcriptome, including 590 with increased m^6^A peaks and 131 with decreased m^6^A peaks (Fig 1A-1C and S1 Table). The top 50 mRNAs with significant increase or decrease in their m^6^A peaks are shown in Fig 1A. The complete distribution of m^6^A sites, regions, and corresponding mRNAs is provided in S1 Table. Among these m^6^A sites, the majority were in the intronic regions (37.6% at 320 sites), distal regions (33.8% at 287 sites), and promoter regions (16.2% at 138 sites) (Fig 1B, 1C and S2 Table). The remaining peaks were distributed across the exonic (non-coding sequence) regions (7.9% at 67 sites), 3’UTR (3.4% at 29 sites), 5’UTR (0.7% at 6 sites), and downstream regions (0.4% at 3 sites) (Fig 1B, 1C and S2 Table). More sites with increased m^6^A methylation than with deceased m^6^A methylation were detected across these regions (Fig 1C). No significant difference in m^6^A peak alterations was observed between the 5’UTR and 3’UTR regions (Fig 1C). Motif analysis identified the top most enriched sequence motifs associated with newly emerged and lost m^6^A peaks in IEC4.1 cells following IFN-γ stimulation (Fig 1D). All sequencing data were generated in accordance with MIAME guidelines and were deposited in the NCBI database (with the NCBI accession numbers: SRR38251195 - SRR38251206).

Gene ontology analysis of mRNAs with altered m^6^A peaks identified a broad range of enriched pathways among both newly gained and lost m^6^A methylation sites. These include immune-related pathways, RNA splicing and translation, mitochondrion functions, and cell proliferation (Fig 1E and S3 Table). Immune-related genes include *Gbp5, Zbp1, Irgm3, Gbp6,* and *Irgm2.* Genes involved in RNA translation and splicing include *Larp1, Pnn, Zfp36, Bcl3,* and *Zrsr2*. Genes associated with mitochondrion functions include *Slc35f6, Gper1, Mmp9,* and *Triap1.* Cell proliferation-related genes included *Batf, Batf3, Tgfa, Has2,* and *B4galt1* (Fig 1E and S3 Table).

**Fig 2:**
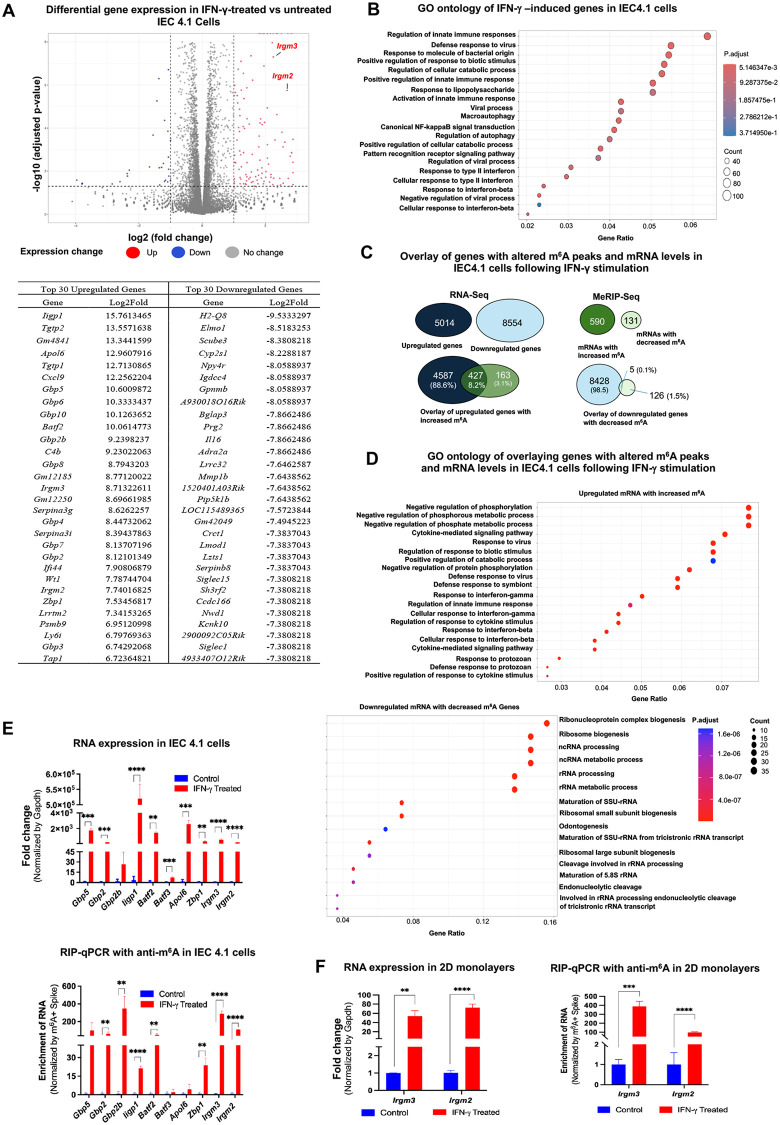
RNA-Seq transcriptomics of intestinal epithelial cells following IFN-γ stimulation. IEC 4.1 cells were treated with IFN-γ (10 ng/mL, 6 h). Total RNAs were collected and processed for whole genome RNA sequencing analysis (RNA-Seq). **(A)** Volcano plot depicting differential gene expression in IFN-γ treated-IEC 4.1 cells vs untreated cells by RNA-Seq. Dashed line represents a cutoff of adjusted p < 0.05. Table lists the top 30 upregulated and downregulated genes after IFN-γ treatment. Two-tailed Wald tests were performed for statistical analysis and *p* value was adjusted by Benjamini-Hochberg method. **(B)** Gene ontology (GO) depicting the biological processes of upregulated genes in IEC4.1 cells following in IFN-γ stimulation. *p* values were calculated based on Kolmogorov-Smirnov test and adjusted by Benjamini-Hochberg method. **(C)** Venn diagram depicting genes with a differential expression level by RNA-seq and with an altered m^6^A level in their mRNAs by m^6^A methylated RNA immunoprecipitation sequence (MeRIP-seq) in cells following IFN-γ stimulation. Overlay of genes represents IFN-γ-induced genes with increased m^6^A status and IFN-γ-downregulated genes with decreased m^6^A status, respectively. (**D)** Gene ontology (GO) depicting the biological processes for overlapped genes induced by IFN-γ with increased m^6^A status. *p* values were calculated based on Kolmogorov-Smirnov test and adjusted by Benjamini-Hochberg method. Data from **A** to **D** were derived from three biological replicates for each group (untreated and IFN-γ treated). (**E)** IFN-γ-induced expression and m^6^A RNA levels of selected immune related genes in IEC4.1 cells by quantitative PCR (qPCR). Cells were treated with IFN-γ (10 ng/mL, 6 h) and total RNAs were collected and processed for qPCR or RIP-qPCR using antibody against m^6^A. (**F)** IFN-γ-induced expression and m^6^A RNA levels of *Irgm2/3* in 2D mouse intestinal epithelial monolayers by qPCR. 2D intestinal epithelial monolayers from mice were treated with IFN-γ (10 ng/mL, 6 h) and total RNAs were collected and processed for qPCR or RIP-qPCR using antibody against m^6^A. Data in **(E** and **F)** are presented as the mean ± standard deviation from three independent experiments and analyzed by Student’s t test; * p < 0.05, ** p < 0.01, ***p < 0.001, **** p < 0.0001.

We also took a portion of the mRNA isolated from untreated and IFN-γ-treated IEC4.1 cells, as described above, for whole genome RNA sequencing (RNA-Seq) analysis. Consistent with previous studies [43], numerous genes were found to be either upregulated or downregulated following IFN-γ stimulation (Fig 2A and S4 Table). The top 30 induced genes are shown in Fig 2A, and a complete list of differentially expressed genes is provided in S4 Table. Among the upregulated genes are immune-related genes (*Gbp2, Irgm3, Irgm2,* and *Zbp1)*, stress-responsive genes (*Psmb9* and *H3c1)*, and metabolism-related genes (*Ido2,* and *Parp14)* (Fig 2B and S5 Table). All sequencing data were generated in accordance with MIAME guidelines and were deposited in the NCBI database (with the GEO accession numbers: GSE245591).

Interestingly, comparison of genes with altered RNA expression and those with changes in m^6^A RNA methylation in IFN-γ-treated cells revealed only a small degree of overlap (Fig 2C and S6 Table). Following IFN-γ stimulation, only 8.2% of genes exhibited both upregulated expression and increased RNA m^6^A levels, while 1.5% showed both downregulated expression and decreased RNA m^6^A levels (Fig 2C and S6 Table). The majority of genes with altered RNA m^6^A levels did not display significant changes in expression following IFN-γ treatment. Representative overlapping genes include *Irgm3, Irgm2, Gbp2b, Zbp1,* and *Batf2* (Fig 2C and S6 Table). Moreover, gene ontology analysis of the 553 genes with both altered expression and m^6^A methylation revealed board biological processes, including cell adhesion, metabolism, and immune responses (Fig 2D and S7 Table). The IFN-γ-induced increase in RNA m^6^A levels in selected genes, and its association with RNA expression, was further validated by quantitative PCR (qPCR) in IEC4.1 cells (Fig 2E) and in 2D mouse intestinal epithelial monolayers (Fig 2F).

### Involvement of Mettl3/14 and lncRNAs in IFN-γ-induced RNA m^6^A in IECs

**Fig 3:**
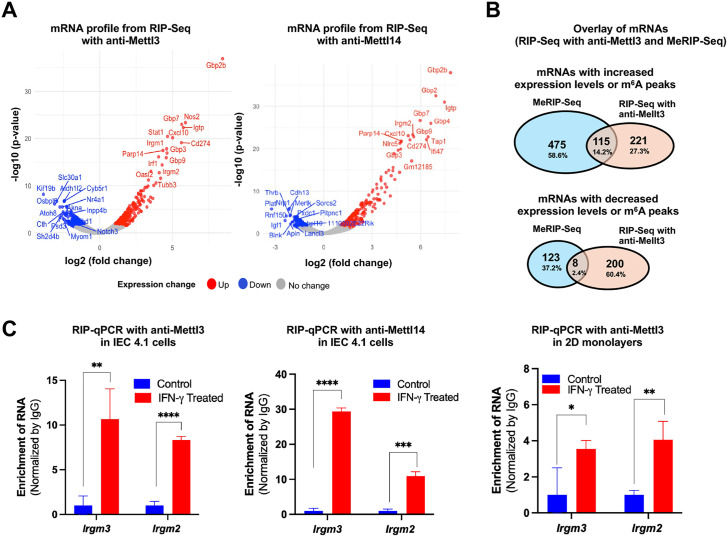
Involvement of Mettl3/14 in IFN-γ-induced RNA m^6^A in intestinal epithelial cells. IEC 4.1 cells were treated with IFN-γ (10 ng/mL, 4 h). Total RNAs were collected and processed for RIP-Seq using antibodies against Mettl3 an Mettl14. **(A)** Volcano plot illustrating differential mRNA profiles from RIP-seq with anti-Mettl3 or RIP-seq with anti-Mettl14 of IFN-γ treated-IEC 4.1 cells vs untreated cells. **(B)** Venn diagram depicting mRNAs associated with Mettl3 and with an altered m^6^A methylation level in cells following IFN-γ stimulation. Overlay of mRNAs represents these that were enriched with anti-Mettl3 and an increased m^6^A level, or with a decreased association with Mettl3 and a decreased m^6^A level. Data (in **A** and **B**) were derived from sequencing of a single biological replicate for each group (untreated and IFN-γ treated). **(C)** IFN-γ-induced association of *Irgm2/3* mRNAs with the Mettl3/14 complex in IEC4.1 cells and 2D mouse intestinal epithelial monolayers by RIP-qPCR. IEC4.1 cells and 2D mouse intestinal epithelial monolayers were exposed to IFN-γ (10 ng/mL) for 4 h and total RNA was collected. Association of *Irgm2* and *Irgm3* mRNAs with the Mettl3/14 complex was assessed by RIP-qPCR with anti-Mettl3 or anti-Mettl14. Data are presented as the mean ± standard deviation from three independent experiments and analyzed by Student’s t test; * p < 0.05, ** p < 0.01, ***p < 0.001, **** p < 0.0001.

RNA m^6^A methylation is primarily mediated by the Mettl3-Mettl14 complex in most eukaryotes [44]. We therefore hypothesized that Mettl3/14 physically associates with target mRNAs to mediate m^6^A methylation. To test this, we performed RIP-Seq using antibodies against Mettl3 and Mettl14 to identify RNAs interacting with these proteins in IEC4.1 cells following IFN-γ treatment. Differential enrichment was analyzed using edgeR, enabling statistical assessment in single-sample comparisons. Our analysis revealed that anti-Mett3 and anti-Mettl14 immunoprecipitation pulled down a similar subset of mRNAs from IFN-γ-treated cells (Fig 3A and S8 Table). Many of these mRNAs overlapped with these exhibiting increased m^6^A methylation following IFN-γ treatment (Fig 3B and S9 Table). The association of Mettl3/14 with selected IFN-γ-induced mRNAs with increased m^6^A methylation (e.g., *Irgm3* and *Irgm2)* was further validated by RNA immunoprecipitation-qPCR (RIP-qPCR) analysis (Fig 3C).

**Fig 4:**
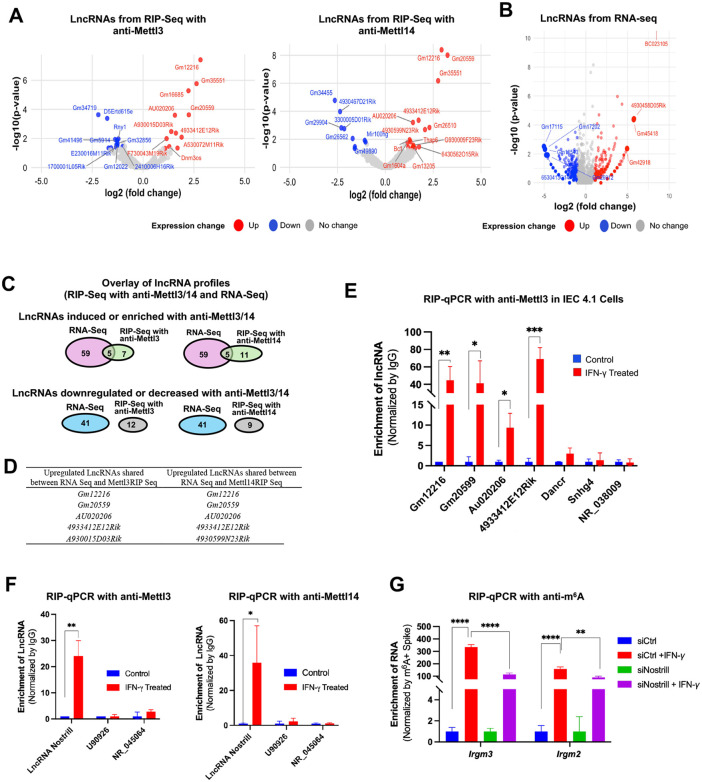
Involvement of lncRNAs in IFN-γ-induced RNA m^6^A in intestinal epithelial cells. **(A)** Volcano plot depicting lncRNAs associated with the Mettl3/14 complex in IFN-γ-treated IEC4.1 cells. Cells were exposed to IFN-γ (10 ng/mL) for 4 h and RNA was collected for RIP-seq with anti-Mettl3 or RIP-seq with anti-Mettl14. Data were derived from sequencing analysis of a single biological replicate for each group (untreated and IFN-γ treated). Differential expression statistics were computed using edgeR enabling statistical testing in single-sample comparisons. **(B)** A volcano plot showing lncRNA expression profiles in IEC4.1 cells following IFN-γ stimulation. Cells were treated with IFN-γ (10 ng/mL) for 4 h and total RNAs were collected for RNA-Seq. Data were derived from sequencing of three biological replicates for each group (untreated and IFN-γ treated). **(C)** Venn diagram depicting lncRNAs with an altered expression levels and associated with the Mettl3/14 complex in IEC4.1 cells following IFN-γ stimulation. Overlay of lncRNAs represents these that were induced with an enriched association with the Mettl3/14 complex, or that were suppressed with a decreased association with Mettl3/14, in cells following IFN-γ stimulation. **(D)** Table listing top 5 lncRNAs with an increased expression level and enriched with the Mettl3/14 complex in IEC4.1 cells following IFN-γ stimulation. **(E)** Validation of IFN-γ-induced enrichment of the top 5 lncRNAs with the Mettl3/14 complex in IEC4.1 cells by RIP-qPCR. Cells were exposed to IFN-γ (10 ng/mL) for 4 h and total RNA was collected. Association of selected lncRNAs with the Mettl3/14 complex was assessed by RIP-qPCR with anti-Mettl3 or anti-Mettl14. Several unrelated lncRNAs, including *Dancr*, *Snhg4*, and *NR_038009*, were used as negative controls. **(F)** IFN-γ-induced enrichment of *Nostrill* with the Mettl3/14 complex in IEC4.1 cells by RIP-qPCR. Cells were exposed to IFN-γ (10 ng/mL) for 4 h followed by RIP-qPCR for *Nostrill* enrichment using anti-Mettl3 or anti-Mettl14. *U90926* and *NR_045064* were used as negative controls. **(G)** Knockdown of *Nostrill* partially blocked IFN-γ-induced m ^6^A methylation of *Irgm3* and *Irgm2* mRNAs in IEC4.1 cells. Cells were transfected with si_Nostrill or si_Control (24 **h)**, then treated with IFN-γ (10 ng/mL, 6h), followed by RIP-qPCR for *Irgm3* and *Irgm2* using anti-m^6^A. Data in (**E, F,** and **G**) are presented as the mean ± standard deviation from three independent experiments and analyzed by Student’s t test; * p < 0.05, ** p < 0.01, ***p < 0.001, **** p < 0.0001.

RIP-Seq using antibodies against Mettl3 and Mettl14 revealed that many lncRNAs were pulled down from IFN-γ-treated cells (Fig 4A and S10 Table). LncRNAs are known to regulate a wide range of cellular functions [45]. RNA-Seq analysis of IEC4.1 cells following IFN-γ stimulation showed significant alterations in the lncRNA expression profile, including 59 upregulated and 41 downregulated lncRNAs (Fig 4C and S11 Table). The top 20 upregulated and downregulated lncRNAs are listed in S12 and S13 Tables. Notably, several lncRNAs identified in the immunoprecipitation with Mettl3 and Mettl14 antibodies were also upregulated in IFN-γ-treated cells, including Gm12216, Gm20599, Au020206, and 4933412E12Rik (Fig 4D and S12 Table). Their association with the Mettl3 protein was further validated by RIP-qPCR (Fig 4E).

We previously demonstrated that induction of the lncRNA Nostrill promotes the expression of *Irgm3* and *iNos* in IECs in response to IFN-γ stimulation [43]. We next investigated whether Nostrill is specifically involved in Mettl3/14-mediated m^6^A methylation of Irgm2/3 following IFN-γ stimulation. RIP-qPCR using anti-Mettl3/14 antibodies confirmed the presence of Nostrill, but not the two unrelated control lncRNAs U90926 and NR_045064, in immunoprecipitates from IFN-γ–treated IEC4.1 cells (Fig 4F). Interestingly, knockdown of Nostrill using siRNA (siR_Nostrill) reduced IFN-γ-induced m^6^A methylation of *Irgm2/3* (Fig 4G). Although Nostrill did not reach statistical significance in the Mettl3/Mettl14 RIP-Seq dataset, this may reflect the limited statistical power of single-sample comparisons and the possibility that not all associated lncRNAs were captured.

### m^6^A RNA methylation modulates IFN-γ-stimulated cell-intrinsic defense against *Cryptosporidium* infection

**Fig 5:**
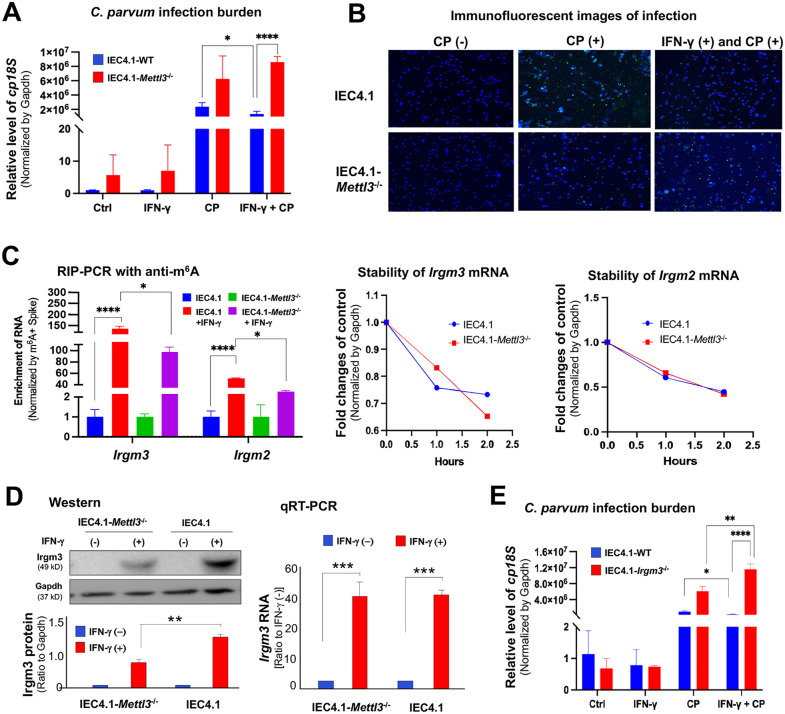
m^6^A RNA methylation modulates IFN-γ-stimulated cell-intrinsic defense against *Cryptosporidium* infection. **(A)** Knockdown of *Mettl3* partially blocked IFN-γ-stimulated epithelial cell-intrinsic defense against *C. parvum* infection. IEC 4.1 and IEC4.1-*Mettl3*^-/-^ cells were exposed to *C. parvum* for 24 h with/or without IFN-γ treatment (5 ng/mL, added at 4 h post infection). *C. parvum* infection burden was measured through qPCR for *Cryptosporidium cp18s* mRNA. **(B)** Representative immunofluorescent staining images of *C. parvum* infection in IEC 4.1 and IEC4.1-*Mettl3*^-/-^ cells from **A.** Blue depicts DAPI while green depicts *C. parvum*. **(C)** m^6^A methylation and RNA stability of *Irgm3* and *Irgm2* in IEC 4.1 and IEC4.1-*Mettl3*^-/-^ cells. Cells were treated with IFN-γ (10 ng/mL, 4 h) followed by RIP-qPCR for *Irgm3* and *Irgm2* using anti-m^6^A. For RNA stability assay, cells were pretreated with IFN-γ (10 ng/mL, 4 h) and then treated with Actinomycin D (10 ug/ml) followed by RNA extraction at 30-minute intervals for a duration of 2 h to quantify RNA expression of *Irgm3* or *Irgm2* by qPCR. **(D)** Knockdown of *Mettl3* partially blocked IFN-γ-induced Irgm3 expression at the protein level but not at the RNA level. IEC 4.1 and IEC4.1-*Mettl3*^-/-^ cells were exposed to IFN-γ (10 ng/mL) for 8 h followed by measurement of Irgm3 at the RNA level (by qPCR) and at the protein level (by Western blot). Densitometric levels of positive bands were quantified and are expressed as the ratio to Gapdh. **(E)** Knockdown of *Irgm3* partially blocked IFN-γ-stimulated cell-intrinsic defense against *C. parvum* infection. IEC 4.1 and IEC4.1-*Irgm3*^-/-^ cells were exposed to *C. parvum* for 24 h with/or without IFN-γ treatment (5 ng/mL, added at 4 h post infection). *C. parvum* infection burden was measured through qPCR for *cp18s.* Data in (**A** to **E**) are presented as the mean ± standard deviation from three independent experiments and analyzed by Student’s t test; * p < 0.05, ** p < 0.01, ***p < 0.001, **** p < 0.0001. CP = *C. parvum*.

We next investigated the effects of inhibiting m^6^A methylation on IFN-γ-stimulated cell-intrinsic antimicrobial defense. To this end, we employed an infection model of IECs with *Cryptosporidium*, a protozoan parasite that infects the gastrointestinal epithelium and other mucosal surfaces in humans and is an important cause of diarrheal disease in young children and AIDS patients [46]. This parasite infects IECs and resides within a specialized intracellular but extracytoplasmic vacuole [47]. IFN-γ-stimulated intrinsic immunity represents a frontline host defense against *Cryptosporidium* infection [48]. Consistent with previous studies [43, 49], we found that treatment of IEC4.1 cells with IFN-γ enhanced cell-intrinsic defense against *Cryptosporidium* infection, as evidenced by a decreased infection burden in the IFN-γ-treated cells compared with untreated controls (Fig 5A). Knockout of Mettl3 in IEC4.1 cells (IEC4.1-*Mettl3*^*-/-*^ cells) using a CRISPR/Cas9 approach (S2 Fig) partially impaired IFN-γ-mediated cell-intrinsic defense, as indicated by a higher infection burden in IFN-γ-treated IEC4.1-*Mettl3*^*-/-*^ cells compared with IFN-γ-treated wild-type cells (Fig 5A and 5B). Consistent with our previous findings [43], treatment of IEC4.1 cells with siR_Nostrill partially inhibited IFN-γ-mediated anti-*Cryptosporidium* defense.

To further investigate the mechanisms by which m^6^A methylation regulates IFN-γ-promoted cell-intrinsic anti-*Cryptosporidium* defense, we examined its effects on the stability of *Irgm2/3* mRNAs in IEC4.1 cells following IFN-γ stimulation. While knockout of *Mettl3* significantly decreased IFN-γ-induced m^6^A methylation of *Irgm2/3* mRNAs, their stability was comparable between IEC4.1 and IEC4.1-*Mettl3*^*-/-*^ cells after IFN-γ treatment (Fig 5C). We next assessed Irgm2/3 protein levels in IEC4.1 and IEC4.1-*Mettl3*^*-/-*^ cells after 8 h of IFN-γ stimulation. Although mRNA levels remained similar between the two groups (Fig 5D), protein levels in IFN-γ-treated IEC4.1 cells were significantly higher than those in IEC4.1-*Mettl3*^*-/-*^ cells exposed to the same amount of IFN-γ (Fig 5D). These findings suggest that m^6^A RNA methylation is required for efficient Irgm2/3 protein expression in IECs in response to IFN-γ stimulation. Importantly, knockout of *Irgm3* in IEC4.1 cells (IEC4.1-*Irgm3*^-/-^ cells) using a CRISPR/Cas9 approach (S2 Fig) partially impaired IFN-γ-mediated cell-intrinsic defense against *Cryptosporidium* infection, as indicated by a higher infection burden in IFN-γ-treated IEC4.1-*Irgm3*^*-/-*^ cells compared with IFN-γ-treated wild-type cells (Fig 5E).

### *Cryptosporidium* infection suppresses *Irgm* RNA m^6^A methylation in infected IECs potentially mediated by parasite-derived CSpV1-dsRNAs

**Fig 6:**
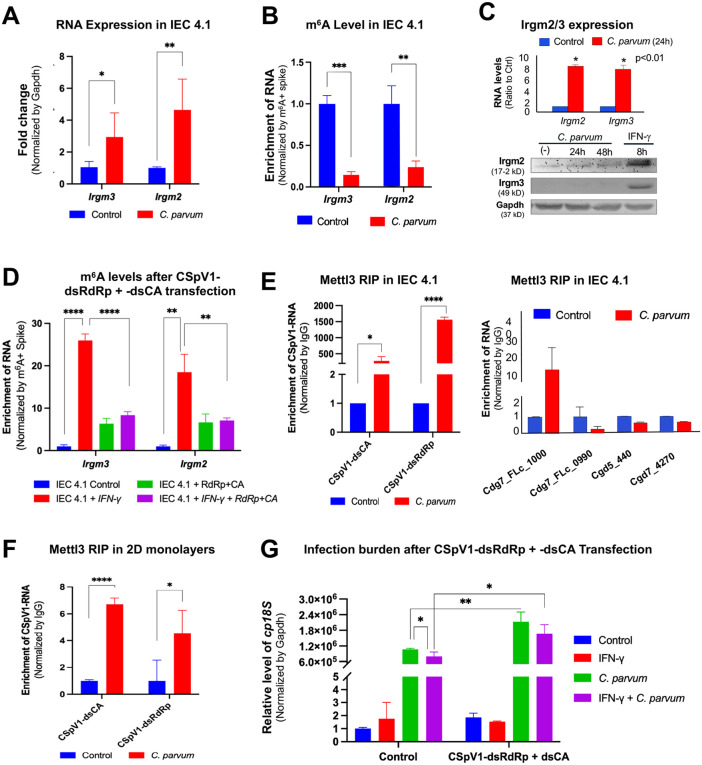
*Cryptosporidium* infection suppresses *Irgm* RNA m^6^A methylation in infected host cells potentially through parasite-derived CSpV1-dsRNAs. **(A)** *C. parvum* infection induces *Irgm3* and *Irgm2* RNA expression in IEC 4.1 cells. Cells were exposed to infection for 24 h and RNA expression of *Irgm3* and *Irgm2* was quantified by qPCR. **(B)** *C. parvum* infection suppresses *Irgm3* and *Irgm2* RNA m^6^A methylation in IEC 4.1 cells. Cells were exposed to infection for 24 h and m^6^A methylation of *Irgm3* and *Irgm2* was measured by RIP-qPCR using antibody to m^6^A. **(C)** *C. parvum* infection causes suppression of Irgm3 and Irgm2 at the protein level in IEC4.1 cells. Cells were exposed to infection for 24 h and 48 **h.** Cell extracts were collected followed by qPCR and Western blot for Irgm2 and Irgm3. Cells treated with IFN-γ for 8 h were used for positive control and Gapdh was also blotted as an internal control of equal loading. Representative gel images were shown. **(D)** Effects of CSpV1-dsRdRp + -dsCA transfection on IFN-γ-induced Irgm2/3 RNA m^6^A methylation in IEC4.1 cells. Cells were transfected with CSpV1-dsRdRp + -dsCA (200 ng each, 24 h) followed by IFN-γ treatment (5 ng/mL, 24h). RNA was collected and processed for RIP-qPCR using anti-m^6^A antibody. **(E)** Association of CSp-V1-dsRNAs with Mettl3 in IEC 4.1 cells following *C. parvum* infection. Cells were infected with *C. parvum* (24 **h)**, RNA was extracted and analyzed by RIP-qPCR using anti-Mettl3 antibody. Presence of CSpV1-dsRdRp and -dsCA, as well as several other RNAs of parasite-origin (*Cdg7_FLc_1000*, *Cdg_FLc_0990*, *Cgd5_440*, and *Cgd_4270*), was assessed using qPCR. **(F)** Association of CSp-V1-dsRNAs with Mettl3 in 2D intestinal epithelial monolayers following *C. parvum* infection. Monolayers were infected with *C. parvum* (24 **h)**, RNA was extracted and analyzed by RIP-qPCR using anti-Mettl3 antibody. **(G)** Effects of CSpV1-dsRdRp + -dsCA transfection on IFN-γ-stimulated cell-intrinsic defense against *C. parvum* infection. IEC4.1 cells were transfected with CSpV1-dsRdRp + -dsCA (200 ng each, 24 **h)**. After transfection, cells were infected with *C. parvum* and IFN-γ was added at 4 h post *C. parvum* infection. Cells were cultured for additional 20 h and RNA was collected, followed by qPCR for *cp18s*. Data **A-G** are presented as the mean ± standard deviation from three independent experiments and analyzed by Student’s t test; * p < 0.05, ** p < 0.01, ***p < 0.001, **** p < 0.0001.

Many pathogens have evolved strategies to evade host immune defense [28]. Similarly, *Cryptosporidium* infection disrupts the IFN-γ signaling pathway in host cells [32], enabling the parasite to evade immune attack during the early stage of infection [30]. In our previous studies we demonstrated that *C. parvum* infection induced significant alterations in m^6^A peaks across 118 regions corresponding to 80 genes in the transcriptome [42]. Specifically, although the RNA levels of *Irgm2* and *Irgm3* were increased in IEC4.1 cells following *C. parvum* infection, m^6^A modifications in their 3’UTRs were reduced in infected cells [42]. Consistently, we observed increased *Irgm2/3* RNA levels (Fig 6A) companied by decreased m^6^A methylation (Fig 6B) in cells following *Cryptosporidium* infection. Interestingly, despite the elevated mRNA levels, Irgm2 and Irgm3 protein levels remained low in infected cells (Fig 6C). These findings suggest that *C. parvum* infection reduces m^6^A methylation of *Irgm2* and *Irgm3* mRNAs, thereby suppressing their protein expression in infected cells.

To elucidate the molecular mechanisms by which *C. parvum* reduces m^6^A methylation of *Irgm2/3*, we investigated whether parasite-derived effectors contribute to this process. In our previous studies, we identified several RNAs with low protein-coding potential in infected host cells [40]. Notably, double-stranded RNAs (dsRNAs) from CSpV1, a member of the family *Partitiviridae* harbored by *C. parvum* and many other *Cryptosporidium* spp, were detected in infected host cells and may modulate host cell function [40]. We therefore examined the m^6^A methylation levels of *Irgm2/3* in IEC4.1 cells following transfection of these parasite-derived RNAs. Transfection with CSpV1-dsRNAs (CSpV1-dsRdRp or CSpV1-dsCA), but not other selected parasite RNAs, resulted in a reduction of m^6^A methylation on *Irgm2/3* transcripts (Fig 6D).

To determine how CSpV1-dsRNAs reduce m^6^A methylation of *Irgm2/3* in IECs, we investigated whether these viral dsRNAs directly interact with *Irgm2/3* mRNAs and thereby interfere with Mettl3/14-mediated m^6^A methylation. RIP assays detected CSpV1-dsRNAs in immunoprecipitates from infected IEC4.1 cells using anti-Mettl3/14 antibodies (Fig 6E). In contrast, such associations between CSpV1-dsRNAs and *Irgm2/3* mRNAs or the Mettl3/14 complex were not observed for other selected parasite-derived RNAs (Fig 6E). Consistently, the interaction between Mettl3/14 and CSpV1-dsRNAs was also detected in 2D intestinal epithelial monolayers following *Cryptosporidium* infection (Fig 6F). Sequence alignment analysis further identified potential complimentary regions between CSpV1-dsRNAs and *Irgm2/3* mRNAs (S3 Fig), supporting the possibility of direct RNA-RNA interactions.

We next investigated the effects of CSPv1-dsRNAs on IFN-γ-stimulated cell-intrinsic antimicrobial defense. Transfection of CSPv1-dsRNAs into IEC4.1 cells partially inhibited IFN-γ-induced m^6^A methylation of *Irgm2/3* mRNAs (Fig 6G). Consistent with this effect, transfection of CSp-V1-dsRNAs also partially impaired IFN-γ-mediated cell-intrinsic defense against *Cryptosporidium* infection, as evidenced by a higher infection burden in IFN-γ-treated IEC4.1 cells transfected with CSp-V1-dsRNAs compared with IFN-γ-treated control cells (Fig 6G).

### m^6^A RNA methylation and IFN-γ stimulated cell-intrinsic anti-*Cryptosporidium* defense in human IECs

**Fig 7:**
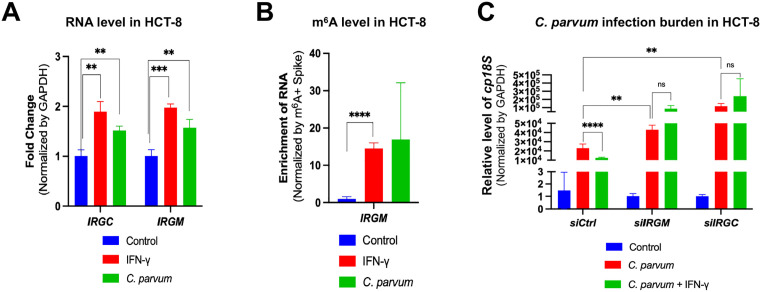
m^6^A methylation of *IRGC* and *IRGM* RNAs and IFN-γ stimulated cell-intrinsic anti-*Cryptosporidium* defense in human HCT-8 cells. **(A)** Expression of human *IRGC* and *IRGM* in HCT-8 cells in response to *C. parvum* infection and IFN-γ stimulation. Cells were exposed to *C. parvum* infection for 6 h or IFN-γ stimulation (10 ng/mL) for 4 h followed by qPCR for *IRGC* and *IRGM*. **(B)** *IRGM* RNA m^6^A methylation in HCT-8 cells in response to *C. parvum* infection and IFN-γ stimulation. Cells were exposed to *C. parvum* infection for 6 h or IFN-γ stimulation (10 ng/mL) for 4 h followed by RIP-qPCR for *IRGM* using anti*-*m^6^A. **(C)** Knockdown of *IRGC* and *IRGM* in HCT-8 cells blocked IFN-γ-stimulated cell-intrinsic defense against *C. parvum* infection. HCT-8 cells were transfected with si_Control, si_IRGM or si_IRGC (25 μM each) for 24 **h.** Cells were then exposed to *C. parvum* for 24 h with/or without IFN-γ treatment (5 ng/mL, added at 4 h post infection). *C. parvum* infection burden was measured through qPCR for *cp18s.* Data in (**A** - **C**) are presented as the mean ± standard deviation from three independent experiments and analyzed by Student’s t test; * p < 0.05, ** p < 0.01, ***p < 0.001, **** p < 0.0001.

We further investigated the role of m^6^A RNA methylation in IFN-γ stimulated cell-intrinsic defense using human intestinal epithelial HCT-8 cells [50]. The human orthologs of murine *Irgm2* and *Irgm3* are *IRGM* and *IRGC,* respectively [14]. We designed PCR primers to assess the expression levels of *IRGC* and *IRGM* in HCT-8 cells. Both genes exhibited only modest increases in expression following IFN-γ stimulation or *C. parvum* infection (Fig 7A). Nevertheless, we observed a significant increase in m^6^A methylation of *IRGM* in IFN-γ-treated cells (Fig 7B), with a similar increasing trend in infected cells (Fig 7B). Consistent with these findings, IFN-γ treatment significantly reduced the infection burden in HCT-8 cells (Fig 7C). Silencing *IRGM* and *IRGC* using siRNAs resulted in a higher infection burden compared with cells treated with a non-specific siRNA control (Fig 7C). Furthermore, knockdown of *IRGM* and *IRGC* largely abolished the IFN-γ-mediated suppression of infection in HCT-8 cells (Fig 7C).

### Discussion

Although m^6^A has recently emerged as an important regulator of host immune responses, the underlying molecular mechanisms remain incompletely defined [51]. Cell-intrinsic defense is critical for controlling *Cryptosporidium*, as a defining feature of infection is the formation of an intracellular yet extracytoplasmic vacuole on the apical surface of infected IECs. Although long-term acquired resistance depends on α/β T cells [52], IFN-γ–mediated epithelial cell-intrinsic immunity is indispensable for early host defense [31]. IFN-γ signaling is well known to induce transcription of numerous ISGs in IECs, many of which encode effector molecules essential for epithelial intrinsic immunity [53]. Our findings indicate that, in addition to transcriptional activation, IFN-γ signaling modulates m^6^A methylation of selective *Irgm* transcripts, thereby contributing to IEC-intrinsic defense against *Cryptosporidium*. Disruption of global m^6^A RNA methylation through Mettl3 knockout diminishes IFN-γ–induced m^6^A modification of *Irgm* transcripts and results in increased *Cryptosporidium* burden in IECs. Thus, regulation of RNA m^6^A methylation following IFN-γ stimulation represents an additional layer of control in epithelial cell-intrinsic antiparasitic defense.

Interestingly, only a small subset of ISGs, such as *Irgm2* and *Irgm3*, exhibits increased m^6^A methylation in IECs following IFN-γ stimulation, suggesting that IFN-γ signaling regulates RNA m^6^A methylation in a gene-specific manner. In most cell types, m^6^A marks are deposited by the METTL3/METTL14 complex, which is recruited to target RNAs in response to specific stimuli [54]. However, the mechanisms governing recruitment of the METTL3/METTL14 complex to distinct RNAs upon activation of different signaling pathways remain poorly understood. Effecter molecules may act as guides to direct METTL3/METTL14 to specific RNAs, thereby enabling gene-specific m^6^A modification. Our data support the possibility that specific lncRNAs serve as such guides for IFN-γ–induced m^6^A methylation of ISGs. LncRNAs are known to function as signals, decoys, guides, or scaffolds by interacting with DNA, RNA, and proteins, and several have been shown in other systems to integrate into the METTL3/METTL14 complex following extracellular stimulation [55–60]. Therefore, lncRNAs may mediate gene-specific recruitment of METTL3/METTL14, enabling selective m^6^A methylation of IFN-γ–responsive ISGs. Among the lncRNAs upregulated in IECs following IFN-γ stimulation, several are physically associated with the METTL3/METTL14 complex, with *Nostrill* being of particular interest. Knockdown of Nostrill disrupts the association of METTL3/METTL14 with *Irgm2* and *Irgm3* transcripts and reduces their IFN-γ-induced m^6^A methylation. Consequently, *Nostrill* knockdown attenuates IFN-γ-mediated cell-intrinsic anti-parasitic defense in IECs.

RNA m^6^A methylation influences multiple aspects of RNA metabolism, including mRNA degradation, splicing, stability, and translation efficiency [60]. Our data suggest that the impact of IFN-γ–induced m^6^A methylation on the stability of ISG transcripts is limited. Instead, m^6^A methylation is closely associated with Irgm2/3 protein expression in IECs following IFN-γ stimulation. Knockout of Mettl3 did not significantly alter *Irgm2/3* RNA levels in IECs in response to IFN-γ stimulation; however, their protein abundance was markedly reduced compared with wild-type cells subject to the same stimulus. Whether this effect of m^6^A RNA methylation on Irgm2/3 protein expression is mediated through regulation of translation and/or protein degradation remains unclear. MeRIP-seq analysis revealed that some IFN-γ–induced m^6^A sites within ISG mRNAs are located near the translation initiation region in the 5′UTRs. The 5′UTR plays a central role in regulating translation initiation, largely through interactions with eukaryotic initiation factor 4E (eIF4E) [61, 62]. The positioning of these m^6^A marks is consistent with a role for m^6^A RNA methylation in modulating translational efficiency. Moreover, knockout of *Irgm3* partially impaired IFN-γ–mediated defense against *Cryptosporidium*, resulting in a significantly higher parasite burden in IFN-γ–treated IEC4.1-*Irgm3 ⁻ / ⁻* cells compared with treated wild-type cells. These findings highlight a critical role for the *Irgm* family members in IFN-γ–stimulated cell-intrinsic defense against *Cryptosporidium*infection.

Notably, we observed a similar role for m^6^A RNA methylation in regulating IFN-γ-mediated cell-intrinsic antiparasitic defense in human IECs. This finding provides new mechanistic insight into species-specific differences in IFN-γ–regulated gene expression, as highlighted in previous studies [63,64]. In particular, transcription of human *IRGM* and *IRGC* is not strongly induced by IFN-γ, in contrast to mice [65, 66]; nevertheless, *IRGM* and *IRGC* remain essential for IFN-γ–mediated control of intracellular pathogens in humans [67]. Our data support a model in which IFN-γ regulates *IRGM/IRGC*-mediated host defense in human IECs through an m^6^A-dependent post-transcriptional mechanism. *IRGM* and *IRGC* are required for epithelial cell-intrinsic defense against *C. parvum* infection in human HCT-8 cells, as knockdown of them impairs IFN-γ–mediated resistance to *Cryptosporidium*. Moreover, increased m^6^A methylation of *IRGM* and *IRGC* transcripts following IFN-γ stimulation supports the notion that IFN-γ modulates *IRGM/IRGC*-mediated host defense through m^6^A-dependent mechanisms in human IECs.

To counteract host defense, *Cryptosporidium* has evolved strategies to disrupt the IFN-γ signaling pathway [32, 34], enabling the parasite to withstand immune pressure during the early stages of infection. In our previous work [40], we demonstrated that CSpV1-dsRNAs are present in infected host cells and attenuate IFN-γ–mediated antiparasitic defense. In the present study, we found that *Cryptosporidium* infection alters m^6^A RNA methylation patterns in IECs, including a reduction in m^6^A methylation on key ISG transcripts such as *Irgm2* and *Irgm3*. Transfection of IECs with CSpV1-dsRNAs recapitulated these effects: CSpV1-dsRNAs reduced m^6^A methylation of ISGs, including *Irgm2* and *Irgm3*, and inhibited the IFN-γ–induced Irgm3 protein expression. Similarly, CSpV1-dsRNA transfection in IEC4.1 cells partially impaired IFN-γ–dependent m^6^A methylation of *Irgm2/3* transcripts and reduced IFN-γ–driven cell-intrinsic defense, as indicated by higher parasite burdens compared with IFN-γ–treated control cells. These findings suggest a previously unrecognized immune evasion strategy: *Cryptosporidium* employs CSpV1-dsRNAs to suppress m^6^A methylation of specific IRG transcripts, thereby undermining IFN-γ–mediated epithelial cell-intrinsic defense. Nevertheless, further studies are needed to clarify the potential involvement of global cellular stress responses induced by infection or dsRNA transfection in regulating RNA m^6^A methylation and translation.

Overall, our findings highlight a new role for m^6^A RNA methylation in IFN-γ–induced anti–*C. parvum* defense, providing new mechanistic insight into the interplay among IFN-γ–mediated epithelial cell-intrinsic defense, m^6^A regulatory machinery, and host antiparasitic immunity. Future investigations should further define how lncRNAs regulate m^6^A RNA methylation on specific ISGs and how these modifications influence the protein expression of individual ISG transcripts, as well as more broadly elucidate the role of m^6^A in IFN-γ–mediated cell-intrinsic immunity.

## Methods

### Ethics statement

This study was carried out in strict accordance with the recommendations in the Guide for the Care and Use of Laboratory Animals of the National Institutes of Health under the Assurance of Compliance Number A3348-01. All animal experiments were done in accordance with procedures (protocol number #23–074) approved by the Institutional Animal Care and Use Committee of Rush University Medical Center.

### Sequencing and bioinformatics

Total RNAs were isolated by Trizol reagent and purified by the RNeasy Mini Kit (QIAGEN 74104). The BGI Americas Corporation (Cambridge, MA) carried out all transcriptome sequencing for RNA-seq, MeRIP-seq, and RIP-Seq with anti-Mettl3/14. For bioinformatics, raw reads were trimmed to remove Truseq adapters and bases from the 3’ end with quality scores less than 20 using cutadapt [68]; trimmed reads shorter than 40 bp were discarded. Trimmed reads were aligned to the Mus musculus (house mouse) genome assembly GRCm39 (mm39) from Genome Reference Consortium [GCA_000001635.9 GCF_000001635.27] using STAR [69]. The expression level of ENSEMBL genes was quantified using FeatureCounts [70]. The bioinformatic analysis of MelRIP-Seq data was performed as described in our previous study [42].

Differential expression statistics was computed using edgeR [71, 72], on raw expression counts obtained from quantification. Normalized expression level was computed as log2 CPM (counts per million), including a TMM normalization and batch effect correction. Comparisons were made between the untreated and IFN-γ-treated groups. In all cases p-values were adjusted for multiple testing using the false discovery rate (FDR) correction of Benjamini and Hochberg [73]. Heatmaps, m^6^A topology charts, gene ontology (GO) analysis, and enhanced volcano plots, were also generated within the R programming language. Predicted RNA-RNA structure analysis was generated using ViennaRNA within the R programming language as well [74, 75]. An absolute LogFC (Log Fold Change) value of greater than 1.5 and *p* value of less than 0.05 were used to generate the volcano plots.

### *C. parvum* and cell lines

*C. parvum* oocysts of the Iowa strain were purchased from a commercial source (Bunch Grass Farm, Deary, ID). The IEC4.1 cell line was a kind gift from Dr. Pingchang Yang (McMaster University, Hamilton, Canada) [41]. Culture media (DMEM/F12 medium 1:1, Fisher Scientific) were supplied with 10% FBS (Ambion) and antibiotics (100 IU/ml of penicillin and 100 µg/ml of streptomycin). HCT-8 cells were acquired from a commercial source (American Type Culture Collection ATCC) and cultured in RPMI 1640 (ATCC), 10% FBS Horse (ATCC), 50 ug/mL Gentamycin (Gibco), with antibiotics (100 IU/ml of penicillin and 100 µg/ml of streptomycin). Stable IEC4.1 cells deficient in *Mettl3* and *Irgm3* were generated through transfection with the CRISPR/Cas9 KO and the HDR plasmids (Santa Cruz Biotechnology), respectively, validated by absence of the corresponding protein, and according to the manufacturer’s instructions and as previously described [76]. The primers used to screen the CRISPR/Cas9 KO cell lines are listed in S13 Table. All cultured cells were maintained at 37°C in a 5% CO_2_ incubator.

### 2D Intestinal epithelial cultures

2D monolayers were isolated and cultured as previously described [77]. Briefly, small intestines were opened longitudinally and washed with ice-cold Ca^2+^ and Mg^2+^ free PBS, then were cut into 1–2 mm fragments and washed with ice-cold Ca^2+^ and Mg^2+^ free PBS 3 times. The cut fragments were incubated in ice-cold 2 mM PBS/EDTA at 4°C for 30min with gentle rotation followed by vigorous shaking until the PBS solution was mostly opaque with dislodged crypt and villus particles. Large tissue fragments were removed through a 100-µm cell strainer (Becton-Dickinson Bioscience, Franklin Lakes, NJ). The pass through was centrifuged 150g for 5 min at 4°C and the pellet was collected as the intestinal epithelium.

### Infection models

For experiments using IEC4.1 and HCT-8 cells or 2D monolayers, viable C*. parvum* oocysts were used. These oocysts were treated with 1% sodium hypochlorite and were added to their respective culture medium. The infection was established by combining oocysts and host cells in a 1:1 ratio. The cell cultures were then incubated at 37°C for 4 h to facilitate parasite attachment and invasion. After this, the cells were thoroughly washed with DMEM-F-12 medium three times to remove any free parasites. The cells were further cultured for varying time periods based on the experimental requirements. qPCR for *Cryptosporidium 18s* (*cp18s*) mRNA and immunofluorescence microscopy were used to assay *C. parvum* infection as previously reported [40]. Primer details are listed in S13 Table.

### qPCR

For quantitative analysis of RNA expression, comparative real-time PCR was performed as previous reported [42, 77] using the SYBR Green PCR Master Mix (Applied Biosystems, Carlsbad, CA). The sequences for all the primers described above are listed in S13 Table.

### siRNAs

Custom-designed RNA oligos against *Nostrill*, *IRGM*, *IRGC*, and a scrambled RNA were synthesized by Sigma. siRNAs were transfected into either IEC4.1 cells or HCT-8 cells with Lipofectamine RNAimax according to the manufacturer’s protocol (Invitrogen). Details, including the sequences of siRNAs, are described in the S13 Table.

### Western blot

Cellular lysate was isolated using the standard approach [78]. Protein concentration of each cell lysate was determined and subsequently analyzed by Western blot. The antibodies anti-Irgm3 (Cell Signaling Technology), anti-Irgm2 (Thermo Scientific), anti-Mettl3 (Abcam), and anti-Gapdh (Santa Cruz Biotechnology) were used for blotting. Precision Plus Protein Dual Color Standards (Bio-Rad) was used as a marker in blotting.

### RNA stability

RNA stability assay was performed by qPCR as previously reported [79]. Briefly, cells were pre-treated with IFN-γ (10 ng/mL, 4 h, R&D System) and transcription was then blocked using actinomycin D (10 µg/ml, Sigma); RNAs were isolated at various time points after actinomycin D treatment. Real-time PCR was then performed using 500 ng of template cDNA for each mRNA gene of interest. Each sample was run in triplicate. The relative abundance of each mRNA was calculated using the ΔΔCt method and normalized to *Gapdh*. The relative amount of mRNA at 0 h following actinomycin D treatment was arbitrarily set to 1. Curve fittings of the resultant data were performed using Microsoft Excel and the half-lives of the RNAs calculated.

### RIP and MeRIP

Formaldehyde crosslinking RIP was performed as described [80]. Briefly, lysates were precleaned with PBS-washed Magna ChIP protein A + G magnetic beads (Millipore). The precleaned lysates were then diluted with whole cell extraction buffer, mixed with specific antibody-coated beads, and then incubated with rotation at 4°C for 4 h. Samples were then washed four times with whole cell extraction buffer containing protease and RNase inhibitors. The collected immunoprecipitated RNP complexes and input were digested in an RNA PK buffer pH 7.0 (100 mM NaCl, 10 mM Tris-HCl pH 7.0, 1 mM EDTA 0.5% SDS) with protease K and incubated at 50°C for 45 min with end-to-end shaking at 400 rpm. Formaldehyde cross-links were reversed by incubation at 65°C with rotation for 4h. RNA was extracted from these samples using Trizol according to the manufacturer’s protocol (Invitrogen) and treated with a DNA-free DNase Treatment and Removal I Kit according to the manufacturer’s protocol (Ambion). The presence of RNA was measured via qPCR. Gene-specific PCR primer pairs are listed in S13 Table. The following antibodies were used for RIP analysis: Normal IgG rabbit (Cell Signaling Technology), anti-Mettl3 (Cell Signaling Technology) and anti-Mettl14 (Cell Signaling Technology)

For MeRIP, total RNAs were isolated by Trizol according to the manufacturer’s protocol (Invitrogen) and m^6^A RIP was performed with the following commercially available kits: Dynabeads mRNA Purification Kit (Invitrogen), NEBNext Magnestium RNA Fragmentation Module Kit (New England Biolabs), Monarch Spin RNA Cleanup Kit (New England Biolabs), and EpiMark N6-Methyladenosine Enrichment Kit (New England Biolabs) that contains a rabbit monoclonal antibody specific for m^6^A.

### Synthesis of CSpV1-dsRNAs and transfection

Synthesis of CSpV1-dsRNAs and transfection was performed as previously described [40]. For specific details of the specific primers and RNA sequences, see S13 Table.

### Statistical analysis

Statistical analysis was performed using edgeR or GraphPad Prism 5 (GraphPad Software). Data are given as mean ± SEM from at least three independent experiments or three biological replicates as indicated. The two-tailed unpaired Student’s *t* test was used for comparisons between two groups. When more than two groups were being compared, one-way ANOVA followed by Tukey’s HSD test was used. The statistical analysis of RNA-Seq, RIP-Seq, and MeRIP-Seq data is described in the Sequencing and Bioinformatics section. *p* values < 0.05 were considered statistically significant.

## Supporting information

S1 Table Full list of mRNAs with increased or decreased m^6^A methylation levels in IEC4.1 cells following IFN-γ stimulation as revealed by MeRIP-Seq using anti-m^6^A antibody. (XLSX)

S2 Table Genomic distribution of m^6^A sites in IEC4.1 cells following IFN-γ stimulation. (XLSX)

S3 Table The GO ontology of mRNAs with significant m^6^A alteration in IEC4.1 cells following IFN-γ stimulation. (XLSX)

S4 Table mRNA gene expression profiles revealed by RNA-Seq in IEC4.1 cells with and without IFN-γ treatment. (XLSX)

S5 Table The GO ontology of mRNAs with significant alterations in their expression in IEC4.1 cells following IFN-γ stimulation. (XLSX)

S6 Table Full list of mRNAs with altered m^6^A peaks and RNA expression levels in IEC4.1 cells following IFN-γ stimulation. (XLSX)

S7 Table The GO ontology of overlapping mRNAs with altered RNA expression and those with changes in m^6^A RNA methylation in IFN-γ-treated IEC4.1 cells. (XLSX)

S8 Table Full list of mRNA profiles revealed by RIP-Seq using anti-Mettl3 and anti-Mettl14 in IEC4.1 cells following IFN-γ stimulation. (XLSX)

S9 Table Full list of mRNAs associated with Mettl3 protein (as revealed by RIP-Seq with anti-Mettl3) and with an altered m^6^A methylation level (as revealed by MeRIP-Seq) in IEC4.1 cells following IFN-γ stimulation. (XLSX)

S10 Table The lncRNA profiles revealed by RIP-Seq using anti-Mettl3 and anti-Mettl14 in IEC4.1 cells following IFN-γ stimulation. (XLSX)

S11 Table The lncRNA gene expression profiles revealed by RNA-Seq in IEC4.1 cells with and without IFN-γ treatment. (XLSX)

S12 Table Full list of lncRNAs associated with the Mettl3/14 complex (as revealed by RIP-Seq with anti-Mettl3 or anti-Mettl13) and with an altered expression level (as revealed by RNA-Seq) in IEC4.1 cells following IFN-γ stimulation. (XLSX)

S13 Table List of PCR primers (used for qPCR, RIP-qPCR, and cloning CSpV1-dsRNAs) and siRNA sequences. (XLSX)

S1 Fig Overlay of mRNAs from RIP-Seq with anti-Mettl14 and MeRIP-Seq in IEC4.1 cells following IFN-γ stimulation. Venn diagram depicting mRNAs associated with Mettl14 and with an altered m^6^A methylation level in IEC4.1 cells following IFN-γ stimulation. Cells were treated with IFN-γ (10 ng/mL, 4 h). Total RNA was collected and processed for MeRIP-Seq using anti-m^6^A antibody. mRNA was used for RIP-Seq using antibodies against Mettl3 an Mettl14. Overlay of mRNAs represents these that were enriched with anti-Mettl14 and an increased m^6^A level, or with a decreased association with Mettl3 and a decreased m^6^A level. Data of MeRIP-Seq were derived from sequencing of three biological replicates for each group (untreated and IFN-γ treated) but data of RIP-Seq with anti-Mettl14 was from a single biological replicate for each group.
(TIF)

S2 Fig Generation of stable IEC4.1 cells with deficient in *Mettl3* or *Irgm3* using the CRISPR/Cas9 approach. Stable IEC4.1 cells with deficient in *Mettl3* or *Irgm3* were generated through transfection of cells with the CRISPR/Cas9 KO and the HDR plasmids for knockout of *Mettl3* and *Irgm3*, respectively. Plasmids were obtained from Santa Cruz Biotechnology. Deletion was verified by using PCR primers covering the designed regions of the genes and by Western blotting.
(TIF)

S3 Fig Predicted RNA - RNA Base Pairing Alignment between CSpV1-dsRNAs and *Irgm2/3* mRNAs for potential direct interactions. Predicted RNA-RNA base pairing alignments between CSpV1-dsRNAs and *Irgm2/3* using RNAduplex, a ViennaRNA package in R to identify favorable intermolecular interactions. Lines indicate complementary base pairing with the local minimum free energy (MFE) shown for the strongest interaction site between the two RNAs.
(TIF)
