# Supplementary figures and images for: m6A RNA methylation modulates IFN-γ-stimulated intestinal epithelial cell-intrinsic antiparasitic defense
Source: PLoS Pathog. 2026 Jul 20;22(7):e1014442. doi: 10.1371/journal.ppat.1014442 (PMC13399507; doi:10.1371/journal.ppat.1014442)

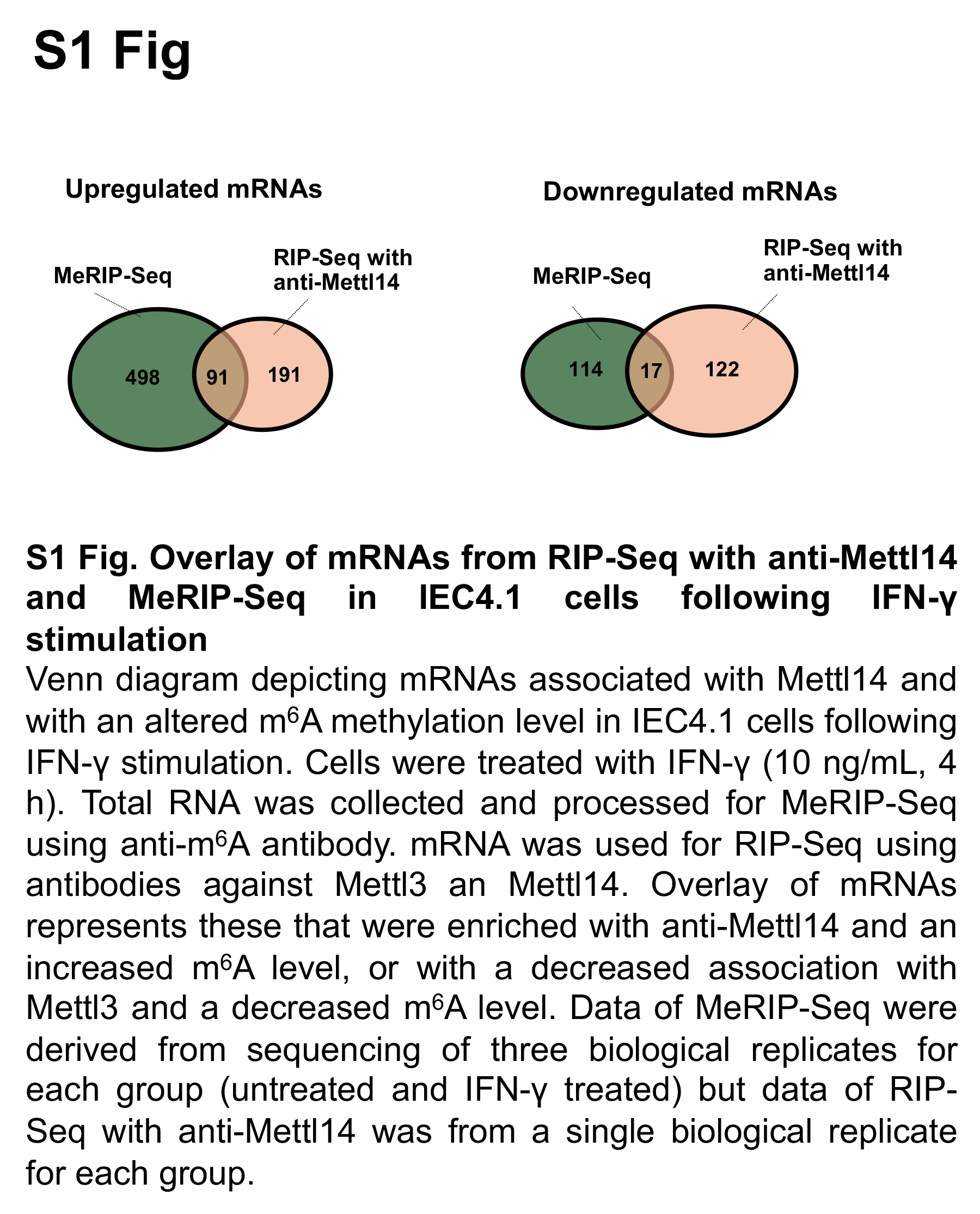

Supplement: S1 Fig — Venn diagram depicting mRNAs associated with Mettl14 and with an altered m6A methylation level in IEC4.1 cells following IFN-γ stimulation. Cells were treated with IFN-γ (10 ng/mL, 4 h). Total RNA was collected and processed for MeRIP-Seq using anti-m6A antibody. mRNA was used for RIP-Seq using antibodies against Mettl3 an Mettl14. Overlay of mRNAs represents these that were enriched with anti-Mettl14 and an increased m6A level, or with a decreased association with Mettl3 and a decreased m6A level. Data of MeRIP-Seq were derived from sequencing of three biological replicates for each group (untreated and IFN-γ treated) but data of RIP-Seq with anti-Mettl14 was from a single biological replicate for each group. (TIF) [file ppat.1014442.s014.tif]

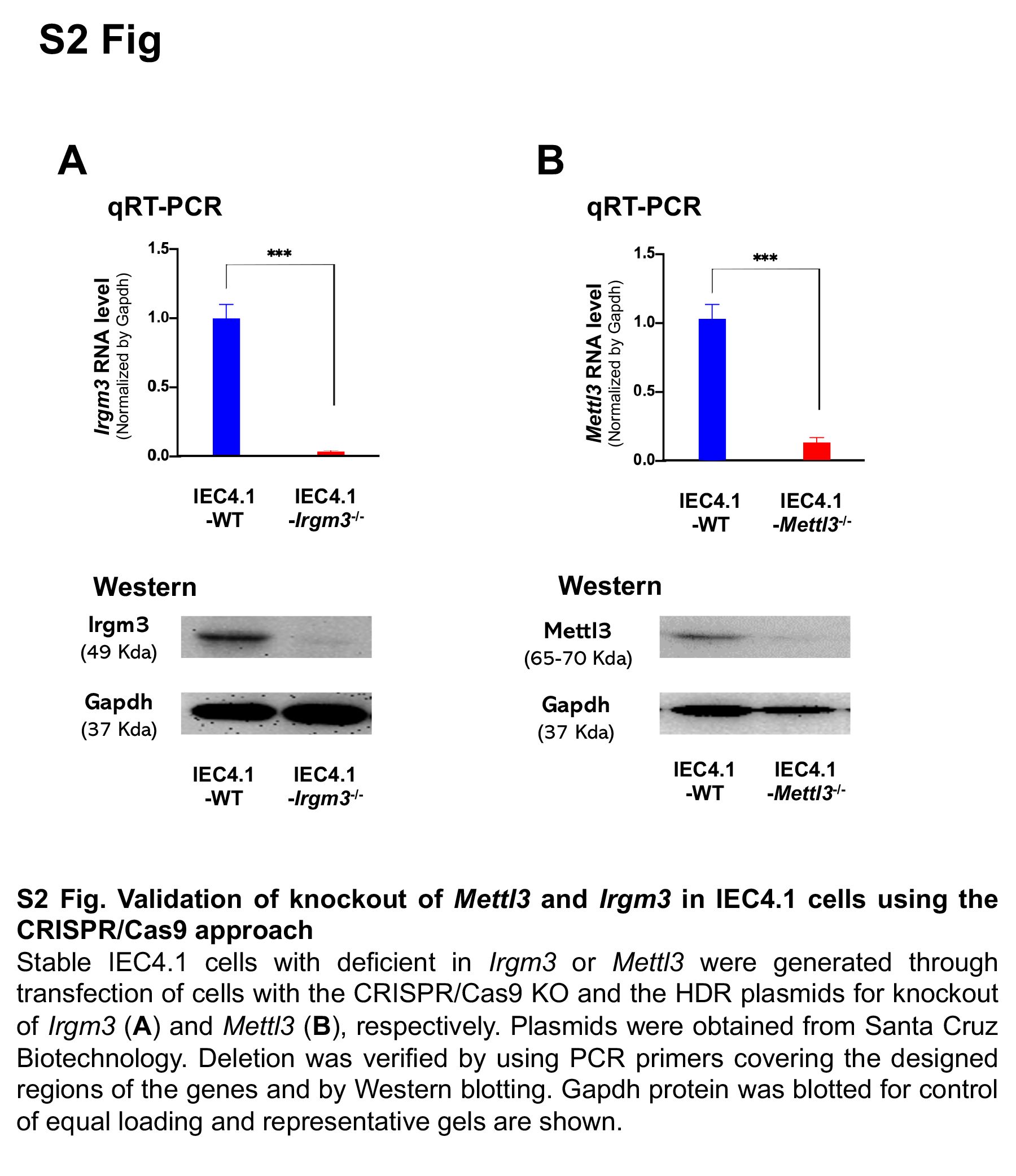

Supplement: S2 Fig — Stable IEC4.1 cells with deficient in Mettl3 or Irgm3 were generated through transfection of cells with the CRISPR/Cas9 KO and the HDR plasmids for knockout of Mettl3 and Irgm3, respectively. Plasmids were obtained from Santa Cruz Biotechnology. Deletion was verified by using PCR primers covering the designed regions of the genes and by Western blotting. (TIF) [file ppat.1014442.s015.tif]

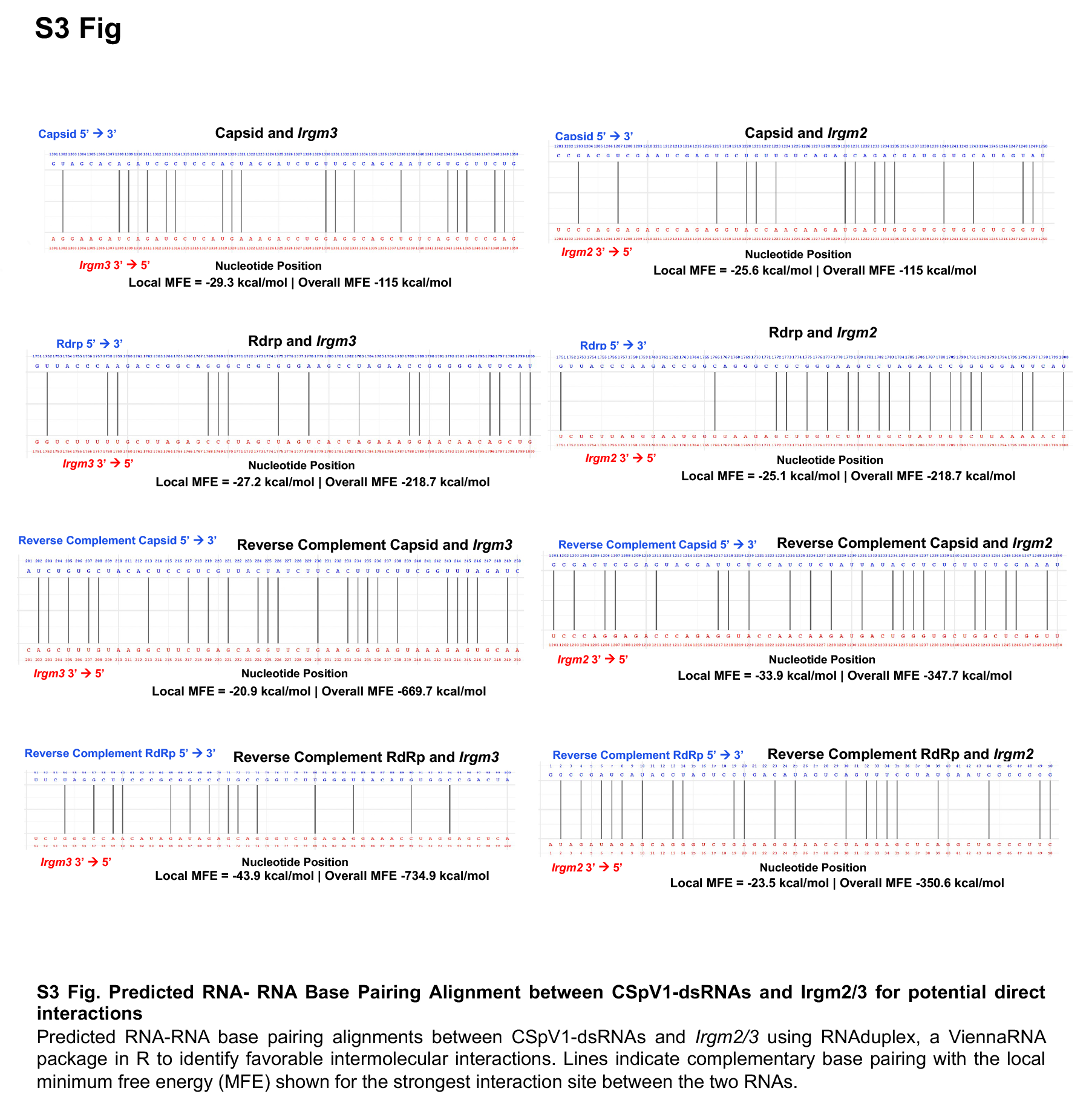

Supplement: S3 Fig — Predicted RNA-RNA base pairing alignments between CSpV1-dsRNAs and Irgm2/3 using RNAduplex, a ViennaRNA package in R to identify favorable intermolecular interactions. Lines indicate complementary base pairing with the local minimum free energy (MFE) shown for the strongest interaction site between the two RNAs. (TIF) [file ppat.1014442.s016.tif]
