# Supplementary material for: m6A RNA methylation modulates IFN-γ-stimulated intestinal epithelial cell-intrinsic antiparasitic defense
Source: PLoS Pathog. 2026 Jul 20;22(7):e1014442. doi: 10.1371/journal.ppat.1014442 (PMC13399507; doi:10.1371/journal.ppat.1014442)
Supplement: Attachment — Submitted filename: Response to Reviewers.docx [file ppat.1014442.s018.docx]

**Point-by-point responses to the reviewers’ comments**

**We thank all the reviewers for your positive comments on our manuscript. We appreciate your thoughtful review and have attempted to satisfactorily respond to your concerns and suggestions.**

**Responses to comments from Reviewer #1**

**Summary**: This study investigates the role of m^6^A RNA methylation in regulating IFN-γ–stimulated intestinal epithelial cell (IEC)–intrinsic defense against *Cryptosporidium parvum*. Using a combination of m^6^A-seq, RNA-seq, RIP-seq, and functional genetic approaches, the authors show that IFN-γ stimulation remodels the m^6^A methylome in IECs and increases m6A modification of a subset of interferon-stimulated genes (ISGs), including Irgm2 and Irgm3. The study further suggests that m^6^A methylation enhances protein output of these transcripts and contributes to epithelial cell-intrinsic anti-parasitic defense. Mechanistically, the authors implicate the METTL3/14 complex and IFN-γ–responsive lncRNAs (notably Nostrill) in directing transcript-specific m^6^A modification, and propose that *Cryptosporidium* infection suppresses this process via delivery of CSpV1-derived dsRNAs. The study addresses an important and timely question at the intersection of RNA epitranscriptomics and host–parasite interactions. The datasets are substantial and generally well-integrated, and the use of complementary approaches—including genetic perturbation and extension to human IEC models—represents a strength. However, several key mechanistic conclusions are stronger than what is directly supported by the data. In particular, the roles of m^6^A in selectively promoting translation, the specificity of lncRNA-guided targeting of the m^6^A machinery, and the proposed direct action of CSpV1-derived dsRNAs would benefit from either additional validation or more cautious interpretation. Addressing these points would improve the rigor and clarity of the study and strengthen its overall impact. ----- We thank you for your positive comments and have modified key mechanistic conclusions with more cautious interpretation of the data to improve the rigor and clarity of the manuscript, details as described below.

**Major issues:** The manuscript is supported by substantial and internally consistent datasets, and the overall conclusions are of interest. The points below focus on key issues that should be addressed to strengthen the central mechanistic claims, primarily through additional analysis or clarification rather than extensive new experimentation.

1). A central conclusion of the study is that m^6^A methylation promotes translation of specific IFN-γ–induced transcripts, such as Irgm2 and Irgm3, thereby contributing to epithelial cell-intrinsic anti-parasitic defense. This interpretation is currently based on the observation that mRNA levels remain comparable while protein abundance is reduced in METTL3-deficient cells. While suggestive, this evidence remains indirect and does not directly assess translational efficiency. To support this key mechanistic claim, the authors should either provide more direct evidence of altered translation (for example, through polysome profiling or other approaches assessing ribosome association), or substantially temper the conclusion and clearly state that the data demonstrate altered protein output without definitively establishing a translational mechanism. ----- We agree with you that the evidence is indirect for the conclusion that m^6^A methylation promotes translation of specific IFN-γ–induced transcripts. To avoid misleading, we have revised the conclusion statement appropriately reflected by the available data in the revised manuscript (lines 16-18, 221-227, 320-327).

2). The manuscript further proposes that CSpV1-derived dsRNAs suppress m^6^A methylation of specific host transcripts by directly interacting with the m^6^A machinery or target RNAs. While the association between dsRNAs and METTL3/14 complexes, together with reduced m^6^A levels, is intriguing, the current data do not establish a direct or specific mechanistic interaction. Alternative explanations, including indirect effects on host RNA metabolism or broader perturbation of cellular processes, are not excluded. To strengthen this aspect of the study, the authors should either provide additional evidence supporting specificity or direct interaction, or more clearly frame this model as a working hypothesis and discuss alternative mechanisms. ----- We thank you for your wonderful insights and suggestions. In the revised manuscript, we have included discussion on alternative mechanisms (lines 18-19, 105-108, 359-363).

3). The manuscript proposes that IFN-γ–induced lncRNAs, particularly Nostrill, guide METTL3/14 to specific transcripts to enable gene-specific m^6^A modification. Although the presented data support an association between Nostrill, METTL3/14, and selected target transcripts, the specificity and generality of this mechanism remain unclear. The authors should either provide additional analysis demonstrating selective targeting of defined transcripts or more explicitly acknowledge the limitations of the current data and moderate claims regarding lncRNA-guided specificity. ----- In the revised manuscript, we have modified associated text to more explicitly acknowledge the limitations of the current data and included more moderate discussion on regarding lncRNA-guided specificity (lines 11-12, 103, 197-199).

**Minor Issues:** The manuscript is generally well organized and presents a substantial amount of data; however, several points could improve clarity, rigor, and presentation. While overall readable, the text would benefit from careful language polishing.

1). There are instances of awkward phrasing (e.g., “control the dynamic of m^6^A RNA modifications” should be “control the dynamics of m^6^A RNA modifications”), minor grammatical issues, and redundancy in some places. ----- We have carefully checked the entire manuscript text to re-word these awkward phrasings, correct these grammatical issues, and decrease redundancy in several places throughout the manuscript.

2). Check consistency in terminology and notation. For example, in m^6^A, “6” is not all in superscript. Gene and protein naming conventions should be standardized to avoid confusion (e.g., Irgm2/3 vs. IRGM/IRGC, and mouse vs. human nomenclature). ----- We have checked the manuscript for consistency in terminology and notation, including standardized gene and protein naming conventions to avoid confusion.

3). In statistical analysis, the use of Student’s t-test and ANOVA is described, but additional details on sample sizes, the definition of biological versus technical replicates, and the application of multiple testing correction (beyond sequencing analyses) should be provided. ----- We have added the information in the revised manuscript (lines 494-499).

4). The biological interpretation of m^6^A peak distribution across genomic regions (e.g., enrichment in intronic, distal, and promoter regions) is not fully discussed (Fig. 1). Additional explanation of how these distributions relate to functional outcomes, particularly translation, can be discussed. ----- We have expanded discussion on the potential impact of m^6^A peak distribution across genomic regions on possible functional outcomes in the revised manuscript (lines 125-134, 325-327).

5). Some figure legends could be expanded, particularly for readers less familiar with epi-transcriptomic analyses. Some terms, such as MeRIP-seq, RIP-seq, and m^6^A peak analyses, may be very briefly explained. ----- We have expanded multiple figure legends to include brief explanation on the related analyses (lines 715-868).

6). In key functional experiments, additional methodological detail would improve confidence. For example, further description of CRISPR/Cas9 knockout validation (e.g., protein-level confirmation and consideration of off-target effects) would be helpful. ----- We have included additional methodological descriptions for these key functional experiments in the revised manuscript (New Fig S2).

7). Some experimental perturbations (e.g., METTL3 knockout, dsRNA transfection, and infection) may induce global cellular stress responses that could influence translation and RNA metabolism. A brief acknowledgment of this possibility in the discussion would provide a more balanced interpretation. ----- We have included this discussion in the revised manuscript (lines 361-363).

**Responses to comments from Reviewer #2**

**Summary**: Pok et al. explore the role of m^6^A RNA methylation in expression of IRG genes including the impact on resistance to *Cryptosporidium*. Using IECs, they find that IFNγ upregulates m^6^A RNA methylation of specific genes including IRG genes. The show that this impacts predominantly translation of the mRNAs rather than mRNA stability. It has previously been shown that IRGs including Irgm3 effectively restrict *C. parvum* growth in IECs. Here it is shown that *C. parvum* can block IFNγ-stimulated m6A RNA methylation of IRGs to greatly reduce their expression. They extend this to some experiments with human IRGM and IRGC. The manuscript is largely well-written and contains novel findings that will be of interest to the research community. ----- We thank you for your positive comments and have revised the manuscript to address all your concerns, as described below.

**Minor Issues:**

1). In the first paragraph of the results, the authors state that m^6^A sites occurred in the promoter region of the RNAs. But promoter regions of genes are generally not transcribed. Please explain this. ----- We have revised the description and included explanation/discussion about this in the manuscript (lines 125-134, 324-327).

2). Fig 1D, the RNA sequence motifs are depicted where m^6^A peaks occur. Why do these RNA sequences have thymines rather than uracils? ----- Sorry that we didn’t convert the DNA sequences from original bioinformatic analysis to RNA motifs. We have corrected this mistake (New Fig 1D).

3). The manuscript is mostly well written, but there are a few sentences that are awkwardly worded. (One example of several is the introductory clause in the second sentence of the 5th paragraph of the Results section). Please edit any such areas. ----- As described above, we have carefully checked the entire manuscript text to re-word these awkward phrasings throughout the whole manuscript, including the introductory clause in the second sentence of the 5th paragraph of the Results section (lines 169-172).

4). In the 7th paragraph of the Results, the authors refer to studies using Nostrill. However, they don’t introduce what Nostrill is. They should supply some basic background regarding Nostrill prior to presenting their data in that section to inform the reader. ----- We have included an introduction of Nostril for our analysis in the revised manuscript (lines 190-191).

5). In several figures in the paper, the authors use discontinuous y-axes. In some cases, this makes relative assessments among the different samples challenging. In some of these instances, it seems that a discontinuous axis really isn’t necessary (including among several Fig. 5A and 5E). Please consider eliminating the discontinuous y-axes when they don’t have to be used. ----- We have eliminated several unnecessary discontinuous y-axes in the revised manuscript (Fig 3C, 4F, 5D, 6A, 6D, 6F, 7A, and 7B).

6). In figure 5D, there are no labels to explain the red and blue bars. ----- We have added labels to explain the red and blue bars in figure 5D.


7). The y-axis in figure 5E does not seem linear. ----- We have corrected the y-axis in figure 5E.

8). The authors state that with *C. parvum* infection, Irgm2 and Irgm3 were not detected. However, there is a faint Irgm2 band in Fig. 6C. Please describe this result accurately. ----- We are sorry for the ambiguous statement regarding Irgm2 and Irgm3 expression and have revised the relevant text in the manuscript (lines 220-226, 240-244, 320-327).

9). In the figure legends, the authors state that the data are averages of three biological replicates. Does this mean that one experiment was performed for each figure with three samples - i.e. the experiments were not repeated? Please clarify. ----- We have included related information in these figure legends in the revised manuscript (lines 715-869).

10). If *C. parvum* completely suppresses Irgm3 expression (Fig. 6C) then why does an Irgm3 knock-out affect *C. parvum* burden (Fig. 5e)? ----- We have included discussions regarding this in the revised manuscript (lines 240-244).

**Responses to comments from Reviewer #3**

**Summary**: Pok et al. have established a role for RNA modifications in the host-pathogen interaction between mammalian intestinal epithelial cells (IECs) and the parasite Cryptosporidium parvum. The authors demonstrate that IEC stimulation with IFNg resulted in changes in patterns of the RNA methylation modification N6-methyladenosine (m^6^A). The m^6^A methylation modification promoted the translation of interferon-stimulated genes (ISGs) such as Irgm3, which supports anti-parasitic functions in IECs. The authors also show that during Cryptosporidium infection, double-stranded RNAs derived from its virus Cryptosporidium parvum virus 1 (CSpV1) suppressed Irg m^6^A methylation and dampened IFNγ-stimulated IEC defense. These data center RNA modifications in the host-pathogen interaction and IFNγ-mediated host defense during parasite infection. These findings are interesting; however, the writing is unclear, resulting in a diffuse narrative that is hard to follow at the outset of the manuscript. In addition, key experimental evidence that supports the authors’ model is lacking. ----- We thank you for your positive comments and have revised the manuscript to address all your concerns, as described below.

**Major issues:**

1). The writing is poor, with grammatical errors and a diffuse narrative, and the manuscript is not clearly constructed. ----- We have carefully checked the entire manuscript text to revise the text for clarity, correct these grammatical errors, and decrease redundancy in several places throughout the entire manuscript.

a. The focus on IRGs does not seem warranted based on the initial text. ----- To strength the rationale of focus on IRGs, we have revised the description regarding the issue in the manuscript (lines 60-64).

b. The description of the first 4 figures is highly descriptive, employing genome-wide assessments to draw conclusions that aren’t experimentally validated as regards their actual function (with the exception of the Nostrill knockdown experiment). While qPCR and other specific readouts are used to validate the genes identified in seq experiments, it’s never clear that the focus will be on IRGs from the text and figures. Some of the data in these figures could be condensed. ----- We agree with you and have revised the description of the first 4 figures. To avoid misleading, we have revised the conclusion/interpretation statement appropriately reflected by the available data in the manuscript.

c. The rationale for studies performed in Fig. 3 is not clear. The statement, “Given the gene specific feature that only a portion the upregulated genes are with an increased m^6^A mRNA level in intestinal epithelial cells upon IFNγ stimulation, we speculate that other mediators, rather than Mett3/14, are the determinants to guide Mett3/14-mediated m^6^A methylation to each targeted mRNA,” is confusing and circular. ----- To avoid misleading and confusing, we have revised the relevant text for better clarity in the manuscript (lines 169-172).

2). The authors do not demonstrate that global, and specifically Irgm2/3 m^6^A, methylation patterns are altered in the MettlKO IEC4.1 cells after IFNγ stimulation. It seems important to validate how this knockout changes Irgm transcript methylation. ----- We totally agree that it is important to validate how the MettlKO in IEC4.1 cells changes Irgm transcript methylation in response to IFNγ stimulation. In our analysis, we detected a significant decrease in the RNA m^6^A methylation of Irgm 2/3 in the MettlKO IEC4.1 cells. However, the knockout didn’t totally block IFNγ-induced Irgm2/3 m^6^A RNA methylation. We have added the data in the manuscript (lines 219-221).

3). Data in Fig. 7 do not close the loop to establish that m^6^A methylation of IRGs is important in human IEC resistance to *Cryptosporidium*, only that IRGs themselves are important and m6A methylation goes up with IFNγ stimulation or infection. ----- We agree with you that the evidence available in human IECs cannot close the loop. To avoid misleading, we have revised the conclusion statement appropriately reflected by the available data in the manuscript (lines 275-284, 345-347).

**Minor Issues:**

1). The abstract is long, diffuse, and contains grammatical errors. It does not clearly convey the results of the study. ----- We have revised the abstract section in the manuscript (lines 13-22).

2). There is no description of the data on the m^6^A motifs altered by IFNγ stimulation in Fig. 1D. ----- We have added description of the data on the m^6^A motifs altered by IFNγ stimulation in Fig. 1D (lines 131-134).

3). The reference that should be to Fig. 1e on page 11, line 17 is to Fig. 1d. ----- We have corrected this mistake.

4). Some of the references for Tables in the text are missing (S2 Table for example). ----- We have added references for tables in the manuscript.

5). There are no scale bars or a key on the figure panel itself for Fig. 5b. ----- We have added scale bars on the figure panel for figure 5b.

6). The Western blot data in Fig. 5d should be quantified. ----- We have included quantified data for the Western blot analysis in Fig 5d (New Fig 5D).

7). The Discussion is lengthy and could be condensed for clarity. ----- We have shortened the discussion section for clarity in the manuscript.
