# Supplementary material for: m6A RNA methylation modulates IFN-γ-stimulated intestinal epithelial cell-intrinsic antiparasitic defense
Source: PLoS Pathog. 2026 Jul 20;22(7):e1014442. doi: 10.1371/journal.ppat.1014442 (PMC13399507; doi:10.1371/journal.ppat.1014442)
Supplement: Peer review history

# Peer review: Decision Letter 0

**DOI:** 10.1371/journal.ppat.1014442.r001

**Authors:** Tracey Lamb

13 Apr 2026

-->PPATHOGENS-D-26-00018

m6A RNA methylation modulates IFN-γ-stimulated intestinal epithelial cell-intrinsic anti-parasitic defense

PLOS Pathogens

Dear Dr. Chen,

Thank you for submitting your manuscript to PLOS Pathogens. After careful consideration, we feel that it has merit but does not fully meet PLOS Pathogens's publication criteria as it currently stands. Therefore, we invite you to submit a revised version of the manuscript that addresses the points raised during the review process.

Please submit your revised manuscript by Jun 12 2026 11:59PM. If you will need more time than this to complete your revisions, please reply to this message or contact the journal office at plospathogens@plos.org. When you're ready to submit your revision, log on to https://www.editorialmanager.com/ppathogens/ and select the 'Submissions Needing Revision' folder to locate your manuscript file.

Please include the following items when submitting your revised manuscript:

* A letter that responds to each point raised by the editor and reviewer(s). You should upload this letter as a separate file labeled 'Response to Reviewers'. This file does not need to include responses to any formatting updates and technical items listed in the 'Journal Requirements' section below.

* A marked-up copy of your manuscript that highlights changes made to the original version. You should upload this as a separate file labeled 'Revised Manuscript with Track Changes'.

* An unmarked version of your revised paper without tracked changes. You should upload this as a separate file labeled 'Manuscript'.

If you would like to make changes to your financial disclosure, competing interests statement, or data availability statement, please make these updates within the submission form at the time of resubmission. Guidelines for resubmitting your figure files are available below the reviewer comments at the end of this letter.

We look forward to receiving your revised manuscript.

Kind regards,

Tracey J. Lamb

Section Editor

PLOS Pathogens

Margaret Phillips

Section Editor

PLOS Pathogens

Sumita Bhaduri-McIntosh

Editor-in-Chief

PLOS Pathogens

orcid.org/0000-0003-2946-9497

Michael Malim

Editor-in-Chief

PLOS Pathogens

orcid.org/0000-0002-7699-2064

**Journal Requirements:**

If the reviewer comments include a recommendation to cite specific previously published works, please review and evaluate these publications to determine whether they are relevant and should be cited. There is no requirement to cite these works unless the editor has indicated otherwise.

1) Please ensure that the CRediT author contributions listed for every co-author are completed accurately and in full.

At this stage, the following Authors/Authors require contributions: Chansorena Pok, Ai-Yu Gong, Marion L. Graham, Shuhong Wang, Silu Deng, Zinat Sharmin, Eugene Lu, Guoqing Lu, Chuan He, and Xian-Ming Chen. Please ensure that the full contributions of each author are acknowledged in the "Add/Edit/Remove Authors" section of our submission form.

The list of CRediT author contributions may be found here: https://journals.plos.org/plospathogens/s/authorship#loc-author-contributions

2) Your manuscript is missing the following sections: Methods. Please ensure all required sections are present and in the correct order. Make sure section heading levels are clearly indicated in the manuscript text, and limit sub-sections to 3 heading levels. An outline of the required sections can be consulted in our submission guidelines here:

https://journals.plos.org/plospathogens/s/submission-guidelines#loc-parts-of-a-submission

3) We note that your Data Availability Statement is currently as follows: "Yes - all data are fully available without restriction". Please confirm at this time whether or not your submission contains all raw data required to replicate the results of your study. Authors must share the “minimal data set” for their submission. PLOS defines the minimal data set to consist of the data required to replicate all study findings reported in the article, as well as related metadata and methods (https://journals.plos.org/plosone/s/data-availability#loc-minimal-data-set-definition).

For example, authors should submit the following data:

1) The values behind the means, standard deviations and other measures reported;

2) The values used to build graphs;

3) The points extracted from images for analysis..

Authors do not need to submit their entire data set if only a portion of the data was used in the reported study.

If your submission does not contain these data, please either upload them as Supporting Information files or deposit them to a stable, public repository and provide us with the relevant URLs, DOIs, or accession numbers. For a list of recommended repositories, please see https://journals.plos.org/plosone/s/recommended-repositories.

If there are ethical or legal restrictions on sharing a de-identified data set, please explain them in detail (e.g., data contain potentially sensitive information, data are owned by a third-party organization, etc.) and who has imposed them (e.g., an ethics committee). Please also provide contact information for a data access committee, ethics committee, or other institutional body to which data requests may be sent. If data are owned by a third party, please indicate how others may request data access.

4) Please amend your detailed Financial Disclosure statement. This is published with the article. It must therefore be completed in full sentences and contain the exact wording you wish to be published.

1) State what role the funders took in the study. If the funders had no role in your study, please state: "The funders had no role in study design, data collection and analysis, decision to publish, or preparation of the manuscript."

2) If any authors received a salary from any of your funders, please state which authors and which funders..

If you did not receive any funding for this study, please simply state: u201cThe authors received no specific funding for this work.u201d

**Reviewers' Comments:**

Reviewer's Responses to Questions

**Part I - Summary**

Please use this section to discuss strengths/weaknesses of study, novelty/significance, general execution and scholarship.

Reviewer #1: This study investigates the role of m6A RNA methylation in regulating IFN-γ–stimulated intestinal epithelial cell (IEC)–intrinsic defense against Cryptosporidium parvum. Using a combination of m6A-seq, RNA-seq, RIP-seq, and functional genetic approaches, the authors show that IFN-γ stimulation remodels the m6A methylome in IECs and increases m6A modification of a subset of interferon-stimulated genes (ISGs), including Irgm2 and Irgm3.

The study further suggests that m6A methylation enhances protein output of these transcripts and contributes to epithelial cell-intrinsic anti-parasitic defense. Mechanistically, the authors implicate the METTL3/14 complex and IFN-γ–responsive lncRNAs (notably Nostrill) in directing transcript-specific m6A modification, and propose that Cryptosporidium infection suppresses this process via delivery of CSpV1-derived dsRNAs .

The study addresses an important and timely question at the intersection of RNA epitranscriptomics and host–parasite interactions. The datasets are substantial and generally well-integrated, and the use of complementary approaches—including genetic perturbation and extension to human IEC models—represents a strength.

However, several key mechanistic conclusions are stronger than what is directly supported by the data. In particular, the roles of m6A in selectively promoting translation, the specificity of lncRNA-guided targeting of the m6A machinery, and the proposed direct action of CSpV1-derived dsRNAs would benefit from either additional validation or more cautious interpretation. Addressing these points would improve the rigor and clarity of the study and strengthen its overall impact.

Reviewer #2: Pok et al. explore the role of m6A RNA methylation in expression of IRG genes including the impact on resistance to Cryptosporidium. Using IECs, they find that IFNg upregulates m6A RNA methylation of specific genes including IRG genes. The show that this impacts predominantly translation of the mRNAs rather than mRNA stability. It has previously been shown that IRGs including Irgm3 effectively restrict C. parvum growth in IECs. Here it is shown that C. parvum can block IFNg-stimulated m6A RNA methylation of IRGs to greatly reduce their expression. They extend this to some experiments with human IRGM and IRGC.

The manuscript is largely well-written and contains novel findings that will be of interest to the research community.

Reviewer #3: Pok et al. have established a role for RNA modifications in the host-pathogen interaction between mammalian intestinal epithelial cells (IECs) and the parasite Cryptosporidium parvum. The authors demonstrate that IEC stimulation with IFNg resulted in changes in patterns of the RNA methylation modification N6-methyladenosine (m6A). The m6A methylation modification promoted the translation of interferon-stimulated genes (ISGs) such as Irgm3, which supports anti-parasitic functions in IECs. The authors also show that during Cryptosporidium infection, double-stranded RNAs derived from its virus Cryptosporidium parvum virus 1 (CSpV1) suppressed Irg m6A methylation and dampened IFNg-stimulated IEC defense. These data center RNA modifications in the host-pathogen interaction and IFNg-mediated host defense during parasite infection. These findings are interesting; however, the writing is unclear, resulting in a diffuse narrative that is hard to follow at the outset of the manuscript. In addition, key experimental evidence that supports the authors’ model is lacking. Specifically:

**********

**Part II – Major Issues: Key Experiments Required for Acceptance**

Please use this section to detail the key new experiments or modifications of existing experiments that should be absolutely required to validate study conclusions.

Generally, there should be no more than 3 such required experiments or major modifications for a "Major Revision" recommendation. If more than 3 experiments are necessary to validate the study conclusions, then you are encouraged to recommend "Reject".

Reviewer #1: The manuscript is supported by substantial and internally consistent datasets, and the overall conclusions are of interest. The points below focus on key issues that should be addressed to strengthen the central mechanistic claims, primarily through additional analysis or clarification rather than extensive new experimentation.

1) A central conclusion of the study is that m6A methylation promotes translation of specific IFN-γ–induced transcripts, such as Irgm2 and Irgm3, thereby contributing to epithelial cell-intrinsic anti-parasitic defense. This interpretation is currently based on the observation that mRNA levels remain comparable while protein abundance is reduced in METTL3-deficient cells. While suggestive, this evidence remains indirect and does not directly assess translational efficiency. To support this key mechanistic claim, the authors should either provide more direct evidence of altered translation (for example, through polysome profiling or other approaches assessing ribosome association), or substantially temper the conclusion and clearly state that the data demonstrate altered protein output without definitively establishing a translational mechanism.

2) The manuscript further proposes that CSpV1-derived dsRNAs suppress m6A methylation of specific host transcripts by directly interacting with the m6A machinery or target RNAs. While the association between dsRNAs and METTL3/14 complexes, together with reduced m6A levels, is intriguing, the current data do not establish a direct or specific mechanistic interaction. Alternative explanations, including indirect effects on host RNA metabolism or broader perturbation of cellular processes, are not excluded. To strengthen this aspect of the study, the authors should either provide additional evidence supporting specificity or direct interaction, or more clearly frame this model as a working hypothesis and discuss alternative mechanisms.

3) The manuscript proposes that IFN-γ–induced lncRNAs, particularly Nostrill, guide METTL3/14 to specific transcripts to enable gene-specific m6A modification. Although the presented data support an association between Nostrill, METTL3/14, and selected target transcripts, the specificity and generality of this mechanism remain unclear. The authors should either provide additional analysis demonstrating selective targeting of defined transcripts or more explicitly acknowledge the limitations of the current data and moderate claims regarding lncRNA-guided specificity.

Reviewer #2: (No Response)

Reviewer #3: 1. The writing is poor, with grammatical errors and a diffuse narrative, and the manuscript is not clearly constructed.

a. The focus on IRGs does not seem warranted based on the initial text.

b. The description of the first 4 figures is highly descriptive, employing genome-wide assessments to draw conclusions that aren’t experimentally validated as regards their actual function (with the exception of the Nostrill knockdown experiment). While qPCR and other specific readouts are used to validate the genes identified in seq experiments, it’s never clear that the focus will be on IRGs from the text and figures. Some of the data in these figures could be condensed.

c. The rationale for studies performed in Fig. 3 is not clear. The statement, “Given the gene specific feature that only a portion the upregulated genes are with an increased m6A mRNA level in intestinal epithelial cells upon IFNg stimulation, we speculate that other mediators, rather than Mett3/14, are the determinants to guide Mett3/14-mediated m6A methylation to each targeted mRNA,” is confusing and circular.

2. The authors do not demonstrate that global, and specifically Irg2/3 m6A, methylation patterns are altered in the MettlKO IEC4.1 cells after IFNg stimulation. It seems important to validate how this knockout changes Irg transcript methylation.

3. Data in Fig. 7 do not close the loop to establish that m6A methylation of IRGs is important in human IEC resistance to Cryptosporidium, only that IRGs themselves are important and m6A methylation goes up with IFNg stimulation or infection.

**********

**Part III – Minor Issues: Editorial and Data Presentation Modifications**

Please use this section for editorial suggestions as well as relatively minor modifications of existing data that would enhance clarity.

Reviewer #1: The manuscript is generally well organized and presents a substantial amount of data; however, several points could improve clarity, rigor, and presentation. While overall readable, the text would benefit from careful language polishing.

1) There are instances of awkward phrasing (e.g., “control the dynamic of m6A RNA modifications” should be “control the dynamics of m6A RNA modifications”), minor grammatical issues, and redundancy in some places.

2) Check consistency in terminology and notation. For example, in m6A, “6” is not all in superscript. Gene and protein naming conventions should be standardized to avoid confusion (e.g., Irgm2/3 vs. IRGM/IRGC, and mouse vs. human nomenclature).

3) In statistical analysis, the use of Student’s t-test and ANOVA is described, but additional details on sample sizes, the definition of biological versus technical replicates, and the application of multiple testing correction (beyond sequencing analyses) should be provided.

4) The biological interpretation of m6A peak distribution across genomic regions (e.g., enrichment in intronic, distal, and promoter regions) is not fully discussed (Fig. 1). Additional explanation of how these distributions relate to functional outcomes, particularly translation, can be discussed.

5) Some figure legends could be expanded, particularly for readers less familiar with epi-transcriptomic analyses. Some terms, such as MeRIP-seq, RIP-seq, and m6A peak analyses, may be very briefly explained.

6) In key functional experiments, additional methodological detail would improve confidence. For example, further description of CRISPR/Cas9 knockout validation (e.g., protein-level confirmation and consideration of off-target effects) would be helpful.

7) Some experimental perturbations (e.g., METTL3 knockout, dsRNA transfection, and infection) may induce global cellular stress responses that could influence translation and RNA metabolism. A brief acknowledgment of this possibility in the Discussion would provide a more balanced interpretation.

Reviewer #2: 1. In the first paragraph of the results, the authors state that m6A sites occurred in the promoter region of the RNAs. But promoter regions of genes are generally not transcribed. Please explain this.

2. Fig 1D, the RNA sequence motifs are depicted where m6A peaks occur. Why do these RNA sequences have thymines rather than uracils?

3. The manuscript is mostly well written, but there are a few sentences that are awkwardly worded. (One example of several is the introductory clause in the second sentence of the 5th paragraph of the Results section). Please edit any such areas.

4. In the 7th paragraph of the Results, the authors refer to studies using Nostrill. However, they don’t introduce what Nostrill is. They should supply some basic background regarding Nostrill prior to presenting their data in that section to inform the reader.

5. In several figures in the paper, the authors use discontinuous y-axes. In some cases, this makes relative assessments among the different samples challenging. In some of these instances, it seems that a discontinuous axis really isn’t necessary. (including among several Fig. 5A and 5E). Please consider eliminating the discontinuous y-axes when they don’t have to be used.

6. In figure 5D, there are no labels to explain the red and blue bars.

7. The y-axis in figure 5E does not seem linear.

8. The authors state that with C. parvum infection, Irgm2 and Irgm3 were not detected. However, there is a faint Irgm2 band in Fig. 6C. Please describe this result accurately.

9. In the figure legends, the authors state that the data are averages of three biological replicates. Does this mean that one experiment was performed for each figure with three samples - i.e. the experiments were not repeated? Please clarify.

10. If C. parvum completely suppresses Irgm3 expression (Fig. 6C) then why does an Irgm3 knock-out affect C. parvum burden (Fig. 5e)?

Reviewer #3: 1. The abstract is long, diffuse, and contains grammatical errors. It does not clearly convey the results of the study.

2. There is no description of the data on the m6A motifs altered by IFNg stimulation in Fig. 1d.

3. The reference that should be to Fig. 1e on page 11, line 17 is to Fig. 1d.

4. Some of the references for Tables in the text are missing (S2 Table for example).

5. There are no scale bars or a key on the figure panel itself for Fig. 5b.

6. The Western blot data in Fig. 5d should be quantified.

7. The Discussion is lengthy and could be condensed for clarity.

**********

PLOS authors have the option to publish the peer review history of their article (what does this mean?). If published, this will include your full peer review and any attached files.

If you choose “no”, your identity will remain anonymous but your review may still be made public.

**Do you want your identity to be public for this peer review?**  For information about this choice, including consent withdrawal, please see our Privacy Policy.

Reviewer #1: No

Reviewer #2: No

Reviewer #3: No

**Figure resubmission:** -->While revising your submission, we strongly recommend that you use PLOS’s NAAS tool (https://ngplosjournals.pagemajik.ai/artanalysis) to test your figure files. NAAS can convert your figure files to the TIFF file type and meet basic requirements (such as print size, resolution), or provide you with a report on issues that do not meet our requirements and that NAAS cannot fix.

After uploading your figures to PLOS’s NAAS tool - https://ngplosjournals.pagemajik.ai/artanalysis, NAAS will process the files provided and display the results in the "Uploaded Files" section of the page as the processing is complete. If the uploaded figures meet our requirements (or NAAS is able to fix the files to meet our requirements), the figure will be marked as "fixed" above. If NAAS is unable to fix the files, a red "failed" label will appear above. When NAAS has confirmed that the figure files meet our requirements, please download the file via the download option, and include these NAAS processed figure files when submitting your revised manuscript.

**Reproducibility:**

To enhance the reproducibility of your results, we recommend that authors of applicable studies deposit laboratory protocols in protocols.io, where a protocol can be assigned its own identifier (DOI) such that it can be cited independently in the future. Additionally, PLOS ONE offers an option to publish peer-reviewed clinical study protocols. Read more information on sharing protocols at https://plos.org/protocols?utm_medium=editorial-email&utm_source=authorletters&utm_campaign=protocols

# Author comment: Author response to Decision Letter 1

**DOI:** 10.1371/journal.ppat.1014442.r002

14 May 2026

Attachment Submitted filename: Response to Reviewers.docx

# Peer review: Decision Letter 1

**DOI:** 10.1371/journal.ppat.1014442.r003

**Authors:** Tracey Lamb

6 Jul 2026

Dear Dr. Chen,

We are pleased to inform you that your manuscript 'm6A RNA methylation modulates IFN-γ-stimulated intestinal epithelial cell-intrinsic antiparasitic defense' has been provisionally accepted for publication in PLOS Pathogens.

Before your manuscript can be formally accepted you will need to complete some formatting changes, which you will receive in a follow up email. A member of our team will be in touch with a set of requests.

Please note that your manuscript will not be scheduled for publication until you have made the required changes, so a swift response is appreciated.

IMPORTANT: The editorial review process is now complete. PLOS will only permit corrections to spelling, formatting or significant scientific errors from this point onwards. Requests for major changes, or any which affect the scientific understanding of your work, will cause delays to the publication date of your manuscript.

Should you, your institution's press office or the journal office choose to press release your paper, you will automatically be opted out of early publication. We ask that you notify us now if you or your institution is planning to press release the article. All press must be co-ordinated with PLOS.

Thank you again for supporting Open Access publishing; we are looking forward to publishing your work in PLOS Pathogens.

Best regards,

Tracey J. Lamb

Section Editor

PLOS Pathogens

Margaret Phillips

Section Editor

PLOS Pathogens

Sumita Bhaduri-McIntosh

Editor-in-Chief

PLOS Pathogens

orcid.org/0000-0003-2946-9497

Michael Malim

Editor-in-Chief

PLOS Pathogens

orcid.org/0000-0002-7699-2064

***********************************************************

Reviewer Comments (if any, and for reference):

Reviewer's Responses to Questions

**Part I - Summary**

Please use this section to discuss strengths/weaknesses of study, novelty/significance, general execution and scholarship.

Reviewer #1: This study investigates the role of m6A RNA methylation in regulating IFN-γ–stimulated intestinal epithelial cell (IEC)–intrinsic defense against Cryptosporidium parvum. Using a combination of m6A-seq, RNA-seq, RIP-seq, and functional genetic approaches, the authors show that IFN-γ stimulation remodels the m6A methylome in IECs and increases m6A modification of a subset of interferon-stimulated genes (ISGs), including Irgm2 and Irgm3.

The study further suggests that m6A methylation enhances protein output of these transcripts and contributes to epithelial cell-intrinsic anti-parasitic defense. Mechanistically, the authors implicate the METTL3/14 complex and IFN-γ–responsive lncRNAs (notably Nostrill) in directing transcript-specific m6A modification, and propose that Cryptosporidium infection suppresses this process via delivery of CSpV1-derived dsRNAs .

The study addresses an important and timely question at the intersection of RNA epitranscriptomics and host–parasite interactions. The datasets are substantial and generally well-integrated, and the use of complementary approaches, including genetic perturbation and extension to human IEC models.

Reviewer #2: Pok et al. explore the role of m6A RNA methylation in expression of IRG genes including the impact on resistance to Cryptosporidium. Using IECs, they find that IFNg upregulates m6A RNA methylation of specific genes including IRG genes. The show that this impacts predominantly translation of the mRNAs rather than mRNA stability. It has previously been shown that IRGs including Irgm3 effectively restrict C. parvum growth in IECs. Here it is shown that C. parvum can block IFNg-stimulated m6A RNA methylation of IRGs to greatly reduce their expression.

**********

**Part II – Major Issues: Key Experiments Required for Acceptance**

Please use this section to detail the key new experiments or modifications of existing experiments that should be absolutely required to validate study conclusions.

Generally, there should be no more than 3 such required experiments or major modifications for a "Major Revision" recommendation. If more than 3 experiments are necessary to validate the study conclusions, then you are encouraged to recommend "Reject".

Reviewer #1: The authors have addressed all major issues in the revised manuscript. I have no more major concerns.

Reviewer #2: The authors have addressed the prior concerns of this reviewer.

**********

**Part III – Minor Issues: Editorial and Data Presentation Modifications**

Please use this section for editorial suggestions as well as relatively minor modifications of existing data that would enhance clarity.

Reviewer #1: All minor concerns have also been addressed. No more concerns.

Reviewer #2: (No Response)

**********

PLOS authors have the option to publish the peer review history of their article (what does this mean?). If published, this will include your full peer review and any attached files.

If you choose “no”, your identity will remain anonymous but your review may still be made public.

**Do you want your identity to be public for this peer review?**  For information about this choice, including consent withdrawal, please see our Privacy Policy.

Reviewer #1: No

Reviewer #2: No

# Editor report: Acceptance letter

**DOI:** 10.1371/journal.ppat.1014442.r004

**Authors:** Tracey Lamb

Dear Dr. Chen,

We are delighted to inform you that your manuscript, "m6A RNA methylation modulates IFN-γ-stimulated intestinal epithelial cell-intrinsic antiparasitic defense," has been formally accepted for publication in PLOS Pathogens.

We have now passed your article onto the PLOS Production Department who will complete the rest of the pre-publication process. All authors will receive a confirmation email upon publication.

The corresponding author will soon be receiving a typeset proof for review, to ensure errors have not been introduced during production. Please review the PDF proof of your manuscript carefully, as this is the last chance to correct any scientific or type-setting errors. Please note that major changes, or those which affect the scientific understanding of the work, will likely cause delays to the publication date of your manuscript. Note: Proofs for Front Matter articles (Pearls, Reviews, Opinions, etc...) are generated on a different schedule and may not be made available as quickly.

Soon after your final files are uploaded, the early version of your manuscript, if you opted to have an early version of your article, will be published online. The date of the early version will be your article's publication date. The final article will be published to the same URL, and all versions of the paper will be accessible to readers.

For Research Articles, you will receive an invoice from PLOS for your publication fee after your manuscript has reached the completed accept phase. If you receive an email requesting payment before acceptance or for any other service, this may be a phishing scheme. Learn how to identify phishing emails and protect your accounts at https://explore.plos.org/phishing.

Thank you again for supporting open-access publishing; we are looking forward to publishing your work in PLOS Pathogens.

Best regards,

Sumita Bhaduri-McIntosh

Editor-in-Chief

PLOS Pathogens

orcid.org/0000-0003-2946-9497

Michael Malim

Editor-in-Chief

PLOS Pathogens

orcid.org/0000-0002-7699-2064
